# The incidence, mortality and complications of systemic lupus erythematosus: a population-level cohort study from 2012 to 2023

**DOI:** 10.1093/rheumatology/keag206

**Published:** 2026-04-17

**Authors:** Samir Patel, Mark D Russell, Katie Bechman, Chris Wincup, Maryam Adas, Deepak Nagra, Alexandru Dregan, Edward Alveyn, Kate Bramham, Sam Norton, James Galloway, Patrick Gordon

**Affiliations:** Centre for Rheumatic Diseases, King’s College London, London, UK; Medical Subspecialties Institute, Cleveland Clinic London, London, UK; Centre for Rheumatic Diseases, King’s College London, London, UK; Centre for Rheumatic Diseases, King’s College London, London, UK; Centre for Rheumatic Diseases, King’s College London, London, UK; Centre for Rheumatic Diseases, King’s College London, London, UK; Department of Basic Medical Sciences, University of Jeddah, Jeddah, Saudi Arabia; Centre for Rheumatic Diseases, King’s College London, London, UK; Centre for Rheumatic Diseases, King’s College London, London, UK; Centre for Rheumatic Diseases, King’s College London, London, UK; Centre for Rheumatic Diseases, King’s College London, London, UK; Medical Subspecialties Institute, Cleveland Clinic London, London, UK; Centre for Rheumatic Diseases, King’s College London, London, UK; Centre for Rheumatic Diseases, King’s College London, London, UK; Centre for Rheumatic Diseases, King’s College London, London, UK

**Keywords:** systemic lupus erythematosus, epidemiology, incidence, mortality, complications, ethnicity

## Abstract

**Objectives:**

Ethnicity-related inequalities in the epidemiology of systemic lupus erythematosus (SLE) have been consistently described, yet robust population-level data remain limited. We aimed to characterize the incidence, all-cause mortality and complications of SLE in a contemporary, ethnically diverse, nationwide cohort.

**Methods:**

We conducted a population-level study using the Clinical Practice Research Datalink in England. Individuals aged ≥18 years with new SLE diagnoses between 2012 and 2023 were included. Controls were selected in a 4:1 ratio and matched on age, sex and primary care practice. Risk of death and complications were estimated using flexible parametric and competing-risk models, respectively.

**Results:**

The cohort consisted of 4937 incident SLE diagnoses (87.9% female; median age 47 years). The incidence of SLE declined over the study period across all ethnicities. Compared with controls (*n* = 19 707), patients with SLE had significantly higher mortality risk [hazard ratio (HR) 2.06; 95% CI: 1.84, 2.32] and elevated risk of all studied complications except solid cancers. SLE incidence was highest among people of Black ethnic background, with the greatest rates observed in Black Caribbean individuals (11.07 per 100 000 person-years in 2023; 95% CI: 6.05, 18.58). Compared with White individuals with SLE, Black individuals had significantly higher mortality (HR 1.64; 95% CI: 1.05, 2.55) and increased risks of diabetes, thrombosis, myocarditis/pericarditis and interstitial lung disease.

**Conclusion:**

Despite declining incidence, mortality for people with SLE was twice that of controls in this large contemporaneous incident cohort. Ethnicity-based inequalities were observed, with elevated mortality and morbidity risk in Black ethnic groups with SLE compared with White ethnic groups.

Rheumatology key messages

- SLE continues to carry substantial mortality and morbidity despite declining incidence.
- Mortality and complication risks appear unevenly distributed across ethnic groups, with the greatest burden identified among those from Black ethnic backgrounds.

## Introduction

Systemic lupus erythematosus (SLE) is characterized by pronounced inequalities in incidence, subsequent complications and potentially mortality [1–4]. Mechanisms remain poorly understood and are likely driven by a complex interplay of genetic predisposition and environmental exposures. As such, contemporary epidemiology heavily influences clinical decision making, research and health policies.

Previous studies have shown varying patterns of mortality and organ damage by ethnicity in people with SLE, with greater renal and cardiovascular disease in those of Black ethnicities [2–8]. However, it remains unclear whether ethnicity coding captures an intrinsic biological risk or instead serves as a surrogate for unmeasured non-biological confounders, such as deprivation, health literacy, healthcare access or structural racism [9].

There have been no reports of SLE incidence by ethnicity in the UK beyond 2012 [1], predating the emergence of new classification criteria [10], greater ethnic diversity in the general English population [11] and biologic therapies. Outside of the USA, very few nationwide studies have described mortality across ethnic groups in diverse settings [4, 8, 12], and none have examined complications at this scale by ethnicity, with research largely derived from specialty clinic-based cohorts such as the Systemic Lupus International Collaborating Clinics (SLICC) group [2, 3]. We therefore sought to address this knowledge gap by utilizing the Clinical Practice Research Datalink (CPRD), one of the world’s largest databases of longitudinal primary care records.

The aim of this study was to explore the current landscape of SLE at a population level in England. Our objectives were to describe the modern incidence, all-cause mortality and complications of SLE by ethnicity using the CPRD.

## Methods

### Data source and study population

We conducted a retrospective population-level observational study using the CPRD Aurum dataset, which contains anonymized data for ∼18 million patients and is broadly representative of the English population in terms of age, sex and ethnicity [13]. Individuals were eligible if they (i) were at least 18 years of age, (ii) had a new SLE diagnosis between 1 January 2012 and 31 December 2023, and (iii) had a minimum of 12 months prior registration with a CPRD-registered primary care practice. This provided a minimum 12-month look-back period within CPRD prior to the index date. We identified patients with incident SLE based on the recording of an index SNOMED code in primary care records for individuals without a previously recorded code [14] (code lists available in Supplementary Table S1). The appearance of a new diagnostic code was considered the index date of diagnosis, aligned with other large epidemiological methodologies [1, 15]. A control cohort matched on age, sex and primary care practice was selected at a four-to-one ratio for every individual with SLE to enable comparative analyses. Controls entered follow-up on the diagnosis date of their matched case and contributed person-time from that date until the earliest of outcome occurrence, death or end of follow-up (31 December 2023).

### Variables and outcomes

Baseline characteristics were extracted for all participants, including age, sex, ethnicity, smoking status, Index of Multiple Deprivation (IMD) quintiles and comorbidities. Ethnicity was self-reported and further refined through linkage with the CPRD Ethnicity Record. The CPRD Ethnicity Record sources underlying data from Hospital Episode Statistics© (HES) were re-used with the permission of The Health & Social Care Information Centre (all rights reserved). We recognized that race and ethnicity are considered separate entities in some countries and acknowledged both as social constructs [16, 17]; however, for consistency and alignment with current reporting [17], we harmonized definitions of a patient’s ethnicity into: White, South Asian, Asian other, Black African, Black Caribbean, Black other, Mixed and Other, and unknown in keeping with broad census categories. Due to low counts and to avoid unstable estimates, ethnicities were consolidated into three categories for mortality and complication analyses: White, Asian and Black.

Date of death was extracted from CPRD. SLE complications of interest were chosen *a priori* based on clinically relevant outcomes analogous to the SLICC damage index (SDI) [18], and likelihood of being captured in primary care records. These included ischaemic heart disease (IHD), heart failure, stroke and transient ischaemic attack (TIA), thrombosis (including venous thromboembolism and arterial thrombosis), myocarditis and/or pericarditis, diabetes, chronic kidney disease (CKD), interstitial lung disease (ILD), osteoporosis, fractures, solid cancers (defined as non-haematological malignancies) and fibromyalgia. For each outcome, we analysed only the first occurrence following SLE diagnosis and excluded individuals with any recorded diagnosis of that outcome prior to the index date, using full CPRD registration history to capture incident events.

Primary care data were collected for renal investigations that are commonly performed in primary care settings: estimated glomerular filtration rates (eGFR), urine protein creatinine ratios (uPCR) and urine albumin creatinine ratios (uACR). In addition to CKD coding, abnormal eGFR, uPCR or uACR measurements present for a minimum of 3 months were used to identify individuals who fulfilled CKD classification criteria as per KDIGO guidance [19].

### Statistical analysis

Incidence, mortality and complication rates were calculated as cases per person-years (py), derived from CPRD and stratified by ethnicity. Calendar year-specific incidence rates were calculated as the number of diagnoses occurring within each calendar year divided by the corresponding person-years at risk accrued during that year. Age and sex-standardized rates were determined using direct standardization to the 2013 European Standard Population [20]. Confidence intervals were estimated using Poisson exact methods. Counts fewer than eight were redacted to ensure statistical disclosure control. Sensitivity analyses were also performed, restricting incident cases to individuals meeting at least one of the following criteria: (i) two SLE SNOMED codes recorded at different timepoints; (ii) one SLE SNOMED code and a disease-modifying anti-rheumatic drug prescription in primary care, or (iii) one SLE SNOMED code and three or more corticosteroid prescriptions in primary care.

Mortality risk was estimated using flexible parametric survival models (Royston–Parmar). We first compared mortality risk between individuals with SLE and matched controls using a model incorporating case/control status and ethnicity as a dummy coded covariate. Models were adjusted for ethnicity and smoking status; age and sex were accounted for through matching. As our primary interest was ethnic differences among individuals with SLE, we then fitted a separate model restricted to the SLE cohort, with ethnicity as the exposure, adjusted for age, sex and smoking status. Time-varying effects of ethnicity on mortality risk were incorporated. Visualization of the hazard functions of survival by ethnicity revealed distinct temporal trends which prompted subdivision of the follow-up period into three intervals: 0–1, 1–5 and >5 years from SLE diagnosis. Hazard ratios of death by ethnicity were estimated for each period, with later intervals reflecting progressively smaller risk sets. Sensitivity analyses were performed separating Black African and Black other groups. Additional analyses exploring assumptions about missing ethnicity showed no meaningful differences and were therefore not presented.

For complication analyses, Fine and Gray models estimated the risk of each outcome in individuals with SLE compared with matched controls, accounting for the competing event of death. Models accounted for the matched design using standard errors clustered at the matched-set level. To determine the risk of complication by ethnicity, analyses were then restricted to the SLE cohort. Models were adjusted for outcome-specific covariates, informed by directed acyclic graphs (Supplementary Fig. S1). Risks were also estimated by age decile and sex in supplementary analyses. Additionally, haematological cancers (lymphoma, leukaemia and myeloma) were examined in supplementary analyses given their distinct association with SLE compared with solid cancers.

### Ethical approval

The CPRD Group has ethical approval from a National Research Ethics Service Committee for all purely observational research using anonymized CPRD data. Scientific approval was given by the CPRD Independent Scientific Advisory Committee for this study (protocol 23_003200).

## Results

Between 1 January 2012 and 31 December 2023, there were 4937 incident diagnoses of SLE contributing 24 900 patient-years of follow-up. Baseline characteristics are presented in Table 1. The age-standardized incidence rate of SLE was 6.6 times higher in females than males over the study period [6.18 (95% CI: 6.00, 6.37) *vs* 0.93 (95% CI: 0.86, 1.01) per 100 000 py]. Women were also younger at diagnosis compared with men [median age at diagnosis: 46 years (IQR 35–58) *vs* 55 years (IQR 43–67)]. Data for age and sex were complete and did not require imputation.

**Table 1:** Baseline characteristics of patients with incident systemic lupus erythematosus (SLE) in the Clinical Practice Research Datalink (CPRD) from 2012 to 2023.

	Total	Male	Female	Diagnosed in 2012	Diagnosed in 2023
	(*n* = 4937)	(*n* = 599)	(*n* = 4338)	(*n* = 443)	(*n* = 376)
Age at diagnosis, median (IQR), years	47.0 (36.0–60.0)	55.0 (43.0–67.0)	46.0 (35.0–58.0)	47.0 (38.0–59.0)	44.0 (33.5–58.0)
Sex, *n* (%)					
Male	599 (12.1)	—	—	53 (12.0)	41 (10.9)
Female	4338 (87.9)	—	—	390 (88.0)	335 (89.1)
Ethnicity, *n* (%)					
White	3509 (71.1)	448 (74.8)	3061 (70.6)	319 (72.0)	234 (62.2)
Mixed/Other	145 (2.9)	11 (1.8)	134 (3.1)	11 (2.5)	19 (5.1)
South Asian	423 (8.6)	59 (9.8)	364 (8.4)	32 (7.2)	38 (10.1)
Asian other	236 (4.8)	26 (4.3)	210 (4.8)	18 (4.1)	30 (8.0)
Black African	253 (5.1)	20 (3.3)	233 (5.4)	17 (3.8)	25 (6.6)
Black Caribbean	171 (3.5)	12 (2.0)	159 (3.7)	24 (5.4)	14 (3.7)
Black other	107 (2.2)	8 (1.3)	99 (2.3)	11 (2.5)	9 (2.4)
Unknown	93 (1.9)	15 (2.5)	78 (1.8)	11 (2.5)	7 (1.9)
IMD decile, *n* (%)					
1 (least deprived)	738 (14.9)	89 (14.9)	649 (15.0)	56 (12.6)	48 (12.8)
2	765 (15.5)	104 (17.4)	661 (15.2)	74 (16.7)	53 (14.1)
3	814 (16.5)	117 (19.5)	697 (16.1)	86 (19.4)	53 (14.1)
4	868 (17.6)	91 (15.2)	777 (17.9)	75 (16.9)	60 (16.0)
5 (most deprived)	872 (17.7)	97 (16.2)	775 (17.9)	70 (15.8)	69 (18.4)
Missing	880 (17.8)	101 (16.9)	779 (18.0)	82 (18.5)	93 (24.7)
Smoking status, *n* (%)					
Non-smoker	2434 (49.3)	200 (33.4)	2234 (51.5)	211 (47.6)	193 (51.3)
Ex-smoker	1578 (32.0)	248 (41.4)	1330 (30.7)	135 (30.5)	126 (33.5)
Current smoker	925 (18.7)	151 (25.2)	774 (17.8)	97 (21.9)	57 (15.2)
Co-morbidities prior to SLE diagnosis, *n* (%)					
Diabetes mellitus	383 (7.8)	66 (11.0)	317 (7.3)	27 (6.1)	31 (8.2)
Hypertension	1114 (22.6)	194 (32.4)	920 (21.2)	101 (22.8)	72 (19.1)
Ischaemic heart disease	284 (5.8)	74 (12.4)	210 (4.8)	21 (4.7)	19 (5.1)
Heart failure	97 (2.0)	26 (4.3)	71 (1.6)	*	*
Stroke/TIA	172 (3.5)	29 (4.8)	143 (3.3)	13 (2.9)	13 (3.5)
CKD stage 3+	433 (8.8)	71 (11.9)	362 (8.3)	37 (8.4)	25 (6.6)
Interstitial lung disease	73 (1.5)	18 (3.0)	55 (1.3)	9 (2.0)	*
Solid cancers	109 (2.2)	12 (2.0)	97 (2.2)	11 (2.5)	*
Osteoporosis	182 (3.7)	14 (2.3)	168 (3.9)	12 (2.7)	12 (3.2)

Baseline characteristics were assessed at the date of SLE diagnosis. Low cell counts (<8) were redacted in accordance with statistical disclosure requirements (*). CKD: chronic kidney disease; IMD: index of multiple deprivation; IQR: interquartile range; TIA: transient ischaemic attack.

There were minimal missing ethnicity data (1.9%), and 71.1% of individuals identified as White. Characteristics stratified by ethnicity are presented in Supplementary Table S2. Individuals of White ethnicity were generally older at diagnosis (median age 50 years; IQR 38–63) compared with other ethnicities. A higher proportion of patients of Black ethnicities were in the most deprived IMD quintile (31.8%) compared with White (14.8%), Mixed/Other (22.8%), South Asian (25.1%) and Asian other (17.4%) patients (Supplementary Table S2).

### Incidence trends over time

**Figure 1:**
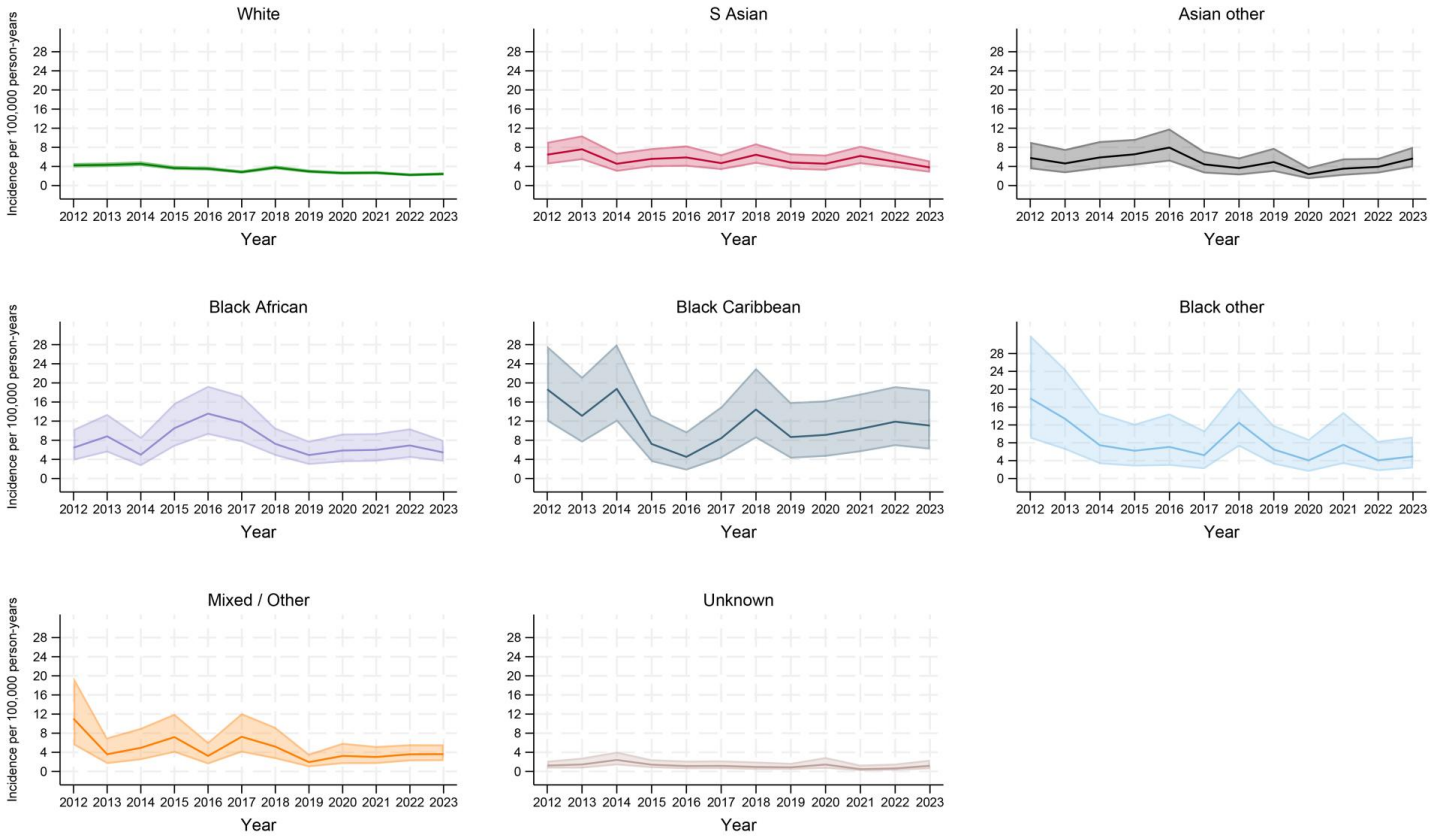
Age and sex-standardized incidence rates of systemic lupus erythematosus (SLE) by ethnic group in the Clinical Practice Research Datalink (CPRD), 2012–2023. Incidence rates are presented per 100 000 person-years with 95% confidence intervals (95% CI), standardized to the 2013 European Standard Population

The age and sex-standardized incidence rate (ASIR) of SLE declined across all ethnic groups during the study period (Fig. 1, Supplementary Table S3). In 2023, the ASIR was highest for those who identified as Black Caribbean (11.07 per 100 000 py; 95% CI: 6.05, 18.58) and lowest for White individuals with SLE (2.42 per 100 000 py; 95% CI: 2.12, 2.76). Using more stringent case definitions resulted in lower ASIR estimates across all ethnic groups; however, temporal trends remained consistent (Supplementary Table S4 and Supplementary Fig. S2).

### All-cause mortality

During the study period, a total of 385 deaths occurred in the SLE cohort and 751 in the control cohort (Table 2). The age and sex-standardized mortality rate (ASMR) within the SLE cohort was 16.74 per 1000 py (95% CI: 15.03, 18.46), more than double that of the control cohort ASMR (7.98 per 1000 py; 95% CI: 7.40, 8.56). Correspondingly, the risk of death for those with SLE was approximately twice as high as matched controls [hazard ratio (HR) 2.06; 95% CI: 1.84, 2.32; Table 3], with minimal variation over time. Men with SLE had 56% higher risk of death compared with women (HR 1.56; 95% CI: 1.23, 1.99; 94 deaths among men, 291 among women). Across all age groups, SLE mortality rates exceeded those of the control group (Supplementary Table S5). The difference was smallest in older age groups [mortality rate ratio 1.61 (95% CI: 1.29, 2.00) for >80-year-olds] and most prominent in younger age groups [mortality rate ratio 5.22 (95% CI: 2.23, 12.21) for 18–39-year-olds], though absolute numbers of deaths were low in 18–39-year-olds.

**Table 2:** Mortality rates in people with systemic lupus erythematosus (SLE) and matched controls, overall and by ethnicity.

	Overall	White	Asian	Black	Unknown
SLE cohort (*n* = 4937)	*n* = 385	*n* = 329	*n* = 21	*n* = 24	*n* = 11
Age at death, median (IQR), years	72.0 (63.0–80.0)	73.0 (65.0–81.0)	70.0 (52.0–72.0)	55.5 (43.0–63.5)	72.0 (66.0–81.0)
Crude mortality rate per 1000 person-years (95% CI)	15.46 (13.99, 17.09)	17.97 (16.08, 20.02)	6.67 (4.13, 10.20)	9.51 (6.09, 14.15)	30.87 (15.41, 55.24)
ASMR per 1000 person-years (95% CI)	16.74 (15.03, 18.46)	16.59 (14.85, 18.49)	13.03 (8.06, 19.91)	11.81 (7.57, 17.58)	31.71 (15.83, 56.74)
Control cohort (*n* = 19 707)	*n* = 751	*n* = 648	*n* = 41	*n* = 17	*n* = 38
Age at death, median (IQR), years	77.0 (68.0–84.0)	78.0 (69.0–84.0)	67.0 (56.0–74.0)	73.0 (62.0–78.0)	75.0 (69.0–82.0)
Crude mortality rate per 1000 person-years (95% CI)	7.71 (7.18, 8.28)	8.25 (7.63, 8.91)	4.54 (3.26, 6.16)	3.48 (2.03, 5.57)	3.66 (1.47, 7.54)
ASMR per 1000 person-years (95% CI)	7.98 (7.40, 8.56)	7.68 (7.10, 8.29)	8.01 (5.75, 10.87)	8.10 (4.72, 12.96)	10.64 (4.28, 21.93)

Crude and age and sex-standardized mortality rates (ASMRs) are presented with 95% CI. Mortality rates are shown overall and stratified by ethnicity. No deaths were observed in the Mixed/Other ethnic group, and therefore this category is not shown. IQR: interquartile range.

**Table 3:** Hazard ratios (HRs) for all-cause mortality in people with systemic lupus erythematosus (SLE) compared with matched controls, and within the SLE cohort by ethnicity.

	Overall	0–1 year from diagnosis	1–5 years from diagnosis	5+ years from diagnosis
SLE *vs* Controls^a^				
SLE	2.06 (1.84, 2.32)	2.02 (1.51, 2.71)	2.06 (1.73, 2.45)	2.08 (1.70, 2.56)
SLE by ethnicity^b^				
White	Reference	Reference	Reference	Reference
Asian	0.99 (0.62, 1.59)	1.29 (0.56, 3.00)	0.89 (0.45, 1.74)	0.66 (0.26, 1.70)
Black	1.64 (1.05, 2.55)	2.21 (1.04, 4.72)	0.98 (0.48, 1.98)	1.47 (0.73, 2.98)

HRs (95% CI) are presented for the overall study period and across time intervals from diagnosis. Estimates with 95% CI were obtained using flexible parametric survival models.

a SLE *vs* Controls: the control population served as the reference group; models were adjusted for ethnicity and smoking status, accounting for age and sex through matching.

b SLE by ethnicity: White ethnicity served as the reference group; models were adjusted for age, sex and smoking status.

### All-cause mortality across ethnic groups

Age at death differed significantly across ethnicities in patients with SLE (*P* < 0.001, Table 2). Median age at death was lowest in Black patients with SLE (55.5 years old; IQR 43.0–63.5) and comparable among other ethnicities (range 70–73 years). No deaths occurred in the Mixed/Other ethnic group. ASMRs appeared higher in all ethnic groups with SLE compared with controls. Within the SLE cohort, the highest ASMR (per 1000 py) was observed for those of unknown (missing) ethnicity (31.71; 95% CI: 15.83, 56.74; 11 deaths) followed by White (16.59; 95% CI: 14.85, 18.49; 329 deaths), Asian (13.03; 95% CI: 8.06, 19.91; 21 deaths) and then Black (11.81; 95% CI: 7.57, 17.58; 24 deaths) ethnicity.

Compared with patients with SLE of White ethnicity, Black ethnicity was associated with a higher risk of death over the whole study period (HR 1.64; 95% CI: 1.05, 2.55; Table 3), but there were no differences between Asian and White ethnicity (HR 0.99; 95% CI: 0.62, 1.59). The excess mortality risk in Black patients appeared greatest in the first year after diagnosis (HR 2.21; 95% CI: 1.04, 4.72), although these time-stratified estimates were based on small event numbers and should be interpreted cautiously. Risk diminished during years 1–5. Beyond 5 years, the hazard ratio increased, although confidence intervals were wide, reflecting smaller numbers at risk and reduced precision in later intervals (Supplementary Fig. S3, Table 3 and Supplementary Table S6). Mortality risk in Asian *vs* White patients with SLE appeared to decline over time, but this was not statistically significant. Sub-categorization of the Black ethnic group revealed the association with death was driven by high mortality among Black African patients (HR 2.11; 95% CI: 1.14, 3.90; Supplementary Table S7; 13 deaths). Counts were too few to estimate hazard ratios by time period within this sensitivity analysis. Upon applying a more stringent SLE definition, the elevated mortality risk among SLE cases compared with controls persisted. Ethnicity-specific mortality hazard ratios were broadly similar, although confidence intervals widened, reflecting reduced precision (Supplementary Table S8).

### Complications

**Figure 2:**
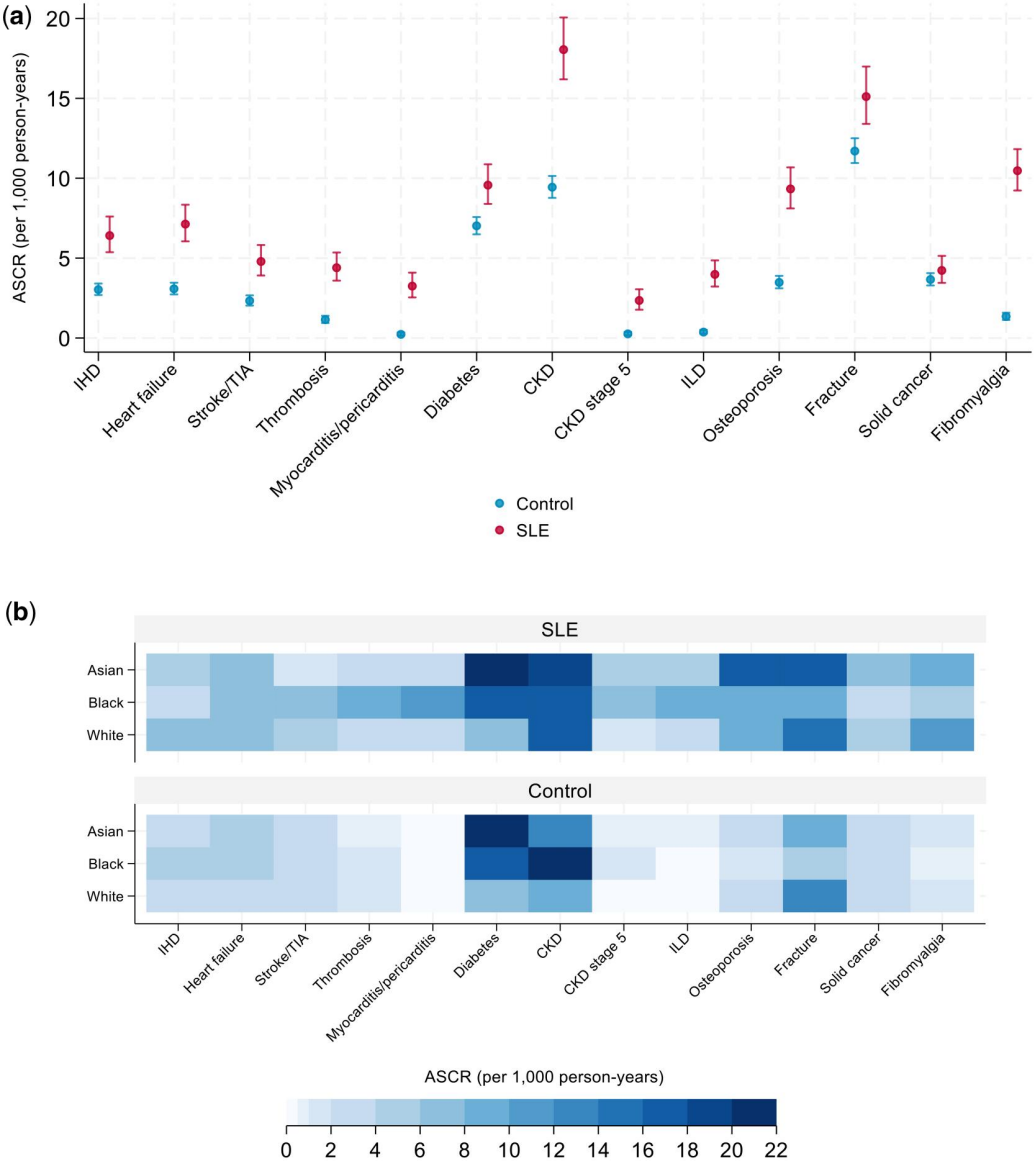
Age-standardized complication rates (ASCRs) for 13 events of interest in people with systemic lupus erythematosus (SLE) and matched controls. (**a**) ASCRs for 13 incident outcomes in the SLE and control cohorts, with 95% confidence intervals represented as bars. (**b**) Heatmap of ASCRs for SLE and control cohorts across studied outcomes, stratified by ethnicity (Asian, Black, White). Confidence intervals are not presented for this panel. CKD: chronic kidney disease; IHD: ischaemic heart disease; ILD: interstitial lung disease; TIA: transient ischaemic attack

**Figure 3:**
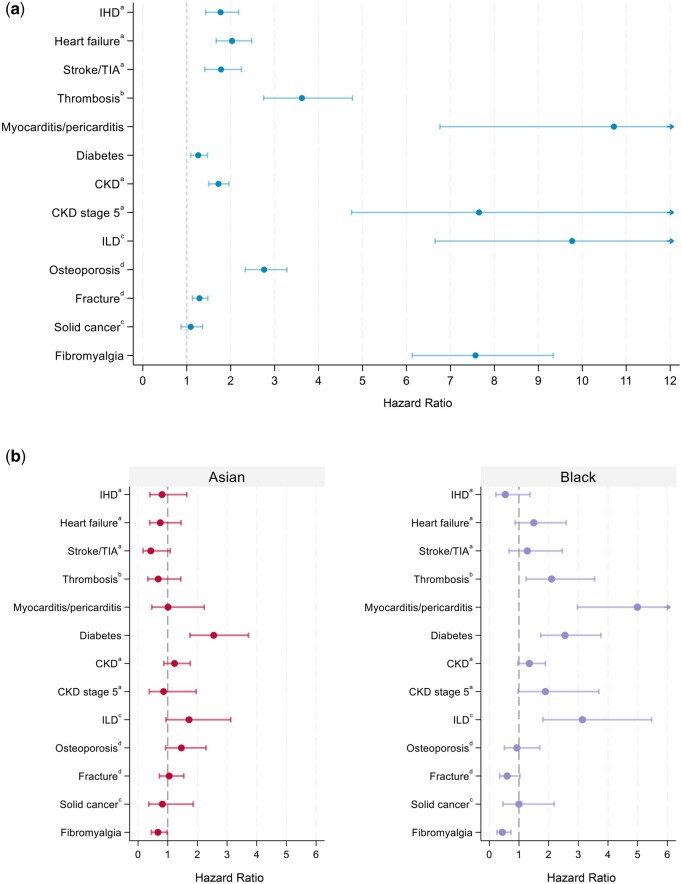
Hazard ratios for developing complications of interest in people with systemic lupus erythematosus (SLE) compared with matched controls. Estimates were obtained using Fine and Gray competing risk models. (**a**) Hazard ratios for incident complications in the SLE cohort compared with matched controls, with 95% confidence intervals represented as bars. All models were adjusted for ethnicity and accounted for age and sex through matching. Additional covariates were selected based on directed acyclic graphs (DAGs). (**b**) Hazard ratios for incident complications in people with SLE from Asian and Black ethnic groups compared with White ethnic groups; 95% confidence intervals represented as bars. Models were adjusted for age and sex, with additional covariates based on DAGs. Additional covariates: ^a^prior diabetes, prior hypertension, smoking status; ^b^smoking status, prior oestrogen use; ^c^smoking status; ^d^alcohol dependence, smoking status. CKD: chronic kidney disease; IHD: ischaemic heart disease; ILD: interstitial lung disease; TIA: transient ischaemic attack

Age-standardized complication rates (ASCRs) were significantly higher for all outcomes in the SLE cohort compared with controls, except for solid cancers (Fig. 2A, Supplementary Tables S9 and S10). Consistent with ASCR findings, patients with SLE had higher risk of developing all complications compared with matched controls, except for solid cancers (Fig. 3A, Supplementary Table S11). The highest risk estimates were seen in myocarditis/pericarditis (HR 10.72; 95% CI: 6.76, 17.01; ASCR 3.25 per 1000 py), ILD (HR 9.77; 95% CI: 6.65, 14.34; ASCR 3.98 per 1000 py), CKD stage 5 (HR 7.65; 95% CI: 4.75, 12.34; ASCR 2.35 per 1000 py) and fibromyalgia (HR 7.57; 95% CI: 6.13, 9.34; ASCR 10.47 per 1000 py). Median time to event was shortest for myocarditis/pericarditis (213 days; IQR 15–772; Supplementary Table S11), thrombosis (625 days; IQR 183–1533), ILD (666 days; IQR 253–1926) and fibromyalgia (710 days; IQR 280–1533). There was a significant inverse relationship between hazard ratios and median time to event (Spearman’s rho −0.72, *P* = 0.007).

Men with SLE were more likely to develop IHD, heart failure, diabetes and CKD stage 5 than women (Supplementary Fig. S4 and Supplementary Table S12) but were less likely to develop osteoporosis or fibromyalgia. Increasing age was associated with elevated risk of IHD, heart failure, stroke/TIA, diabetes, CKD, ILD, osteoporosis, fractures and solid cancers within the SLE cohort (Supplementary Fig. S5 and Supplementary Table S13). Younger age was associated with a heightened risk of myocarditis/pericarditis, CKD stage 5 and fibromyalgia in people with SLE. Haematological cancers were observed more frequently and were associated with a higher risk in the SLE cohort compared with controls (HR 2.36; 95% CI: 1.53, 3.64; ASCR 1.53 per 1000 py, Supplementary Table S14). Counts were too few for ethnicity-stratified analyses.

### Complications across ethnic groups

Within the SLE cohort, Black patients had higher rates of diabetes, thrombosis, myocarditis/pericarditis, CKD stage 5 and ILD (Fig. 2B, Supplementary Table S15). On multivariate analysis, Black ethnicity was associated with higher estimated risks of diabetes (HR 2.55; 95% CI: 1.73, 3.76), thrombosis (HR 2.10; 95% CI: 1.24, 3.55), myocarditis/pericarditis (HR 4.99; 95% CI: 2.97, 8.39) and ILD (HR 3.14; 95% CI: 1.81, 5.47) compared with White ethnicity (Fig. 3B, Supplementary Table S16). Although the hazard ratio for myocarditis/pericarditis and ILD in Black compared with White patients was highest in the first year after SLE diagnosis, confidence intervals overlapped with later periods and differences between intervals should therefore be interpreted cautiously (Supplementary Table S17). Asian patients with SLE were more frequently diagnosed with diabetes and osteoporosis than White patients with SLE, but only diabetes remained significantly associated with Asian ethnicity in adjusted models (HR 2.55; 95% CI: 1.75, 3.72).

## Discussion

This study represents one of the largest recent epidemiological reports of SLE using multiple standardized methods to demonstrate a disproportionate burden of SLE across ethnic groups. While SLE outcomes are universally poor, our findings suggest the presence of ethnicity-based inequalities in both complications and mortality that require urgent attention.

Individuals with newly diagnosed SLE had substantially higher rates and risk of death compared with age and sex-matched controls, underscoring the severity of this condition. Our findings are consistent with a previously reported crude mortality rate of 15.84 per 1000 py in a UK incident SLE cohort from 1999 to 2012 [21]. Further to this, we provided mortality data for a larger cohort by ethnicity, with age-standardization and multivariate regression analyses, which suggested higher risk in those of Black ethnicities.

Accordingly, all complication rates and risks were higher in patients with SLE compared with controls, except for solid cancers. In general, long-term complications such as those associated with atherosclerosis (IHD, heart failure, stroke), metabolic dysfunction (diabetes, osteoporosis) and CKD (due to atherosclerosis and metabolic dysfunction) showed relatively modest hazard ratios (1.26 for diabetes to 2.76 for osteoporosis). These long-term complications likely reflect the latent pathophysiology of SLE, including accelerated atherosclerosis [22], indirect effects (e.g. reduced physical activity) and treatment-related sequelae (e.g. corticosteroid-induced hyperglycaemia, insulin resistance and osteoporosis). In contrast, thrombosis, myocarditis/pericarditis and ILD were associated with higher hazard ratios (ranging from 3.62 to 10.72) and shorter median times to onset. These more acute complications are probably driven by inadequately suppressed inflammation around the time of diagnosis.

Before our report, no studies had described the rate or risk of ILD, myocarditis or pericarditis in patients with SLE on a population level in England. Conrad *et al.* described an increased risk of developing myocarditis and pericarditis in those with a variety of autoimmune diseases (including SLE) compared with controls [23]. However, heterogeneity of conditions in this group was high, for example SLE combined with type 1 diabetes. Similarly, Gonnelli *et al.* reported the incidence of connective tissue disease-related ILD (CTD-ILD) [24], but no specific inference could be drawn for SLE-related ILD. Importantly, the absence of SLE-specific screening recommendations in the recently published European CTD-ILD guidelines reflects the limited evidence on ILD risk factors in SLE [25]. In this context, our findings add to a sparse literature, but should be interpreted cautiously, as ILD diagnoses were based on clinician-recorded codes from routinely collected data.

Two prior studies using the CPRD GOLD dataset reported rates and risk of cardiovascular disease, heart failure, stroke, renal disease, diabetes, cancer and osteoporosis between 1997 and 2013 [26, 27]. Kuo *et al.* found hazard ratios comparable to ours, except for renal diseases (adjusted HR 3.43, compared with 1.72 for CKD in our study) and diabetes (adjusted HR 0.99, compared with 1.26 in our study) [27]. They identified renal diseases based on READ codes alone and included acute conditions, for example acute renal tubular necrosis, whereas we incorporated laboratory tests to aid CKD detection.

Our exploratory findings suggested ethnicity-based inequalities, whereby Black ethnicity was associated with higher overall risk of mortality compared with White ethnicity. This was particularly evident in the first year from diagnosis and was also observed in those of Black African ethnicity on subgroup analyses. These findings are consistent with our recent meta-analysis, where Black and Indigenous patients with SLE had higher odds of death compared with White patients [4]. While pooled odds ratios from five non-USA-based studies in this meta-analysis showed no significant mortality difference between Black and White ethnicities, none were population-based studies.

In contrast to the higher mortality risk observed for Black patients compared with White patients with SLE, ASMRs showed no significant ethnic differences. This may be explained by age structure confounding (Black patients in our SLE cohort had a comparatively younger median age at diagnosis and death); time-varying effects of ethnicity on death that were not captured by ASMRs; or the increased ability of flexible parametric survival models to detect differences between groups. The higher mortality risk among Black patients compared with White patients with SLE is complex and likely combines both biological and non-biological factors. We observed higher risks of thrombosis, myocarditis/pericarditis and ILD in Black patients compared with White patients with SLE, which are potentially key contributors to mortality. As these conditions are clinically evident and objectively diagnosed, ethnicity-based differences presumably reflect true relationships rather than miscoding or health-seeking behaviour bias. Previous studies have reported increased pericarditis risk in Black patients compared with White patients with SLE (Hopkins Lupus cohort) [28] and higher myocarditis odds in Black *vs* Hispanic patients with SLE (LUMINA cohort) [29]. No comparable studies examining thrombosis or ILD by ethnicity in patients with SLE were found.

Non-biological factors play a central role in the heightened risk of death for patients with SLE and may explain some of the observed high risk in Black patients. These include socioeconomic deprivation, health literacy inequities and structural racism. Any of these reasons may lead to diagnostic or treatment delays and early mortality. Crucially, Black patients with SLE resided in more disadvantaged IMD areas than White patients in our study, suggestive of greater deprivation (Supplementary Table S2). Outside of the UK, Ward *et al.* suggested that socioeconomic status (private/public insurance and median household income), not race, was associated with mortality in SLE [30]. This highlights a key challenge in epidemiological analysis of ethnic inequalities, as socioeconomic status may be considered both a mediator and an independent risk factor. Arguably, as ethnicity is a social construct and not entirely representative of genetic admixture or ancestry, socioeconomic factors may be the key driver. However, attempting to accurately estimate an individual’s socioeconomic status is problematic as it symbolizes an intangible concept shaped by nuance, exclusion, among other factors—not simply a function of income, education, health literacy or postcodes. As such, we did not include IMD in our models.

This report also suggested a gradual decline in the recorded incidence of SLE across all ethnicities from 2012 to 2023, although estimates for smaller groups were based on fewer observations, particularly among individuals with missing ethnicity data. The highest incidence was consistently seen in those of Black ethnicities (Black Caribbean, Black African, Black other), in keeping with Rees *et al.* [1]. However, compared with our analysis and reflective of electronic health records at the time, they identified a smaller cohort of incident cases (*n* = 1643) and had a higher proportion of missing ethnicity data (25%). The higher incidence of SLE in those of Black or Asian ethnicities has an unclear aetiology. Theories regarding ancestry-related genetic predispositions [31, 32] and environmental evolutionary pressures, such as endemic parasitic infections or high ultraviolet light exposure [33, 34], have merit but are unsubstantiated.

The main strengths of this study are the breadth of data and outcomes examined. In one report, we provide the first population-level estimates of mortality and complications by ethnicity from an incident SLE cohort outside the USA, together with contemporary incidence rates by ethnicity in the UK. Notably, this study provides the first population-level estimates of clinician-recorded ILD, myocarditis/pericarditis rates and risks in English patients with SLE. Methodologically, our approach examined outcomes against matched controls and within the SLE cohort itself, revealing overall disease burden and intrinsic inequalities. The use of flexible parametric survival models enabled exploration of evolving hazards of death over time by ethnicity, and we accounted for competing risk bias in our statistical models for SLE complications.

A key limitation of using electronic health records is reliance on accurate coding during consultations, which may vary over time with changes in practice and data capture. Primary care physicians are unlikely to attribute SLE codes to patients without specialist review, although the potential for diagnostic misclassification remains. Differences in solid cancer outcomes may reflect incomplete ascertainment of certain subtypes, long latency periods and the competing risk of death. We were also unable to robustly capture some SLE-specific complications, such as lupus nephritis; ascertain the chronicity of certain complications to calculate a proxy SDI; or determine cause-specific deaths. Follow-up was censored at practice deregistration, which may reflect migration or transfer of care; individuals entering the cohort later in the study therefore had shorter available follow-up, and although loss to follow-up was broadly similar across major ethnic groups, differential censoring cannot be excluded. Therefore, we did not interpret crude temporal patterns in complications or mortality and instead used survival analyses that appropriately account for censoring. Low event counts were redacted to ensure stable estimates and reduce disclosure risk, limiting precision and granularity for some ethnic subgroups, leading to exploratory analyses. Finally, we acknowledge the risk of surveillance bias and relative under-reporting of complications among controls, though use of a population-level data source helps to mitigate this concern.

## Conclusion

Although SLE incidence has declined, people with SLE remain approximately twice as likely to die and face higher morbidity than controls. Using primary care data, we observed a greater burden among individuals from Black ethnic backgrounds, including higher early mortality and associations with diabetes, thrombosis, myocarditis/pericarditis and ILD compared with people from White ethnic backgrounds. These findings raise concern that ethnicity-based inequities in outcomes may limit the benefits of modern SLE therapies in real-world practice.

## Supplementary Material

keag206_Supplementary_Data

## Data Availability

All data relevant to the study are included in the article or uploaded as supplementary information.
