# Supplementary material for: The incidence, mortality and complications of systemic lupus erythematosus: a population-level cohort study from 2012 to 2023
Source: Rheumatology (Oxford). 2026 Apr 17;65(5):keag206. doi: 10.1093/rheumatology/keag206 (PMC13183668; doi:10.1093/rheumatology/keag206)
Supplement: keag206_Supplementary_Data [file keag206_supplementary_data.pdf]

## Supplementary material

Supplementary Figures ..... 3

Supplementary Figure 1. Directed acyclic graphs (DAGs) for outcomes of interest. ..... 3

Supplementary Figure 2. Age and sex-standardised incidence rates of systemic lupus erythematosus (SLE) in the Clinical Practice Research Datalink (CPRD) using stringent case-definition criteria. Rates shown by ethnicity and year, 2012-2023. ..... 9

Supplementary Figure 3. Risk of death following diagnosis of systemic lupus erythematosus (SLE) over time. ..... 10

Supplementary Figure 4. Risk of developing complications of interest in men compared with women. ..... 11

Supplementary Figure 5. Association between age and risk of developing complications of interest in people with systemic lupus erythematosus (SLE), with age modelled as a continuous variable in deciles. ..... 12

Supplementary Tables ..... 13

Supplementary Table 1. SNOMED-CT code lists used to define systemic lupus erythematosus (SLE), comorbidities, and complications of interest. ..... 13

Supplementary Table 2. Baseline characteristics of patients with incident systemic lupus erythematosus (SLE) by ethnicity in the Clinical Practice Research Datalink (CPRD) from 2012 to 2023. ..... 14

Supplementary Table 3. Age and sex-standardised incidence rates (ASIRs) of systemic lupus erythematosus (SLE) in the Clinical Practice Research Datalink (CPRD) by ethnicity and year, 2012-2023, with 95% confidence intervals (95% CI). ..... 15

Supplementary Table 4. Age and sex-standardised incidence rates (ASIRs) of systemic lupus erythematosus (SLE) in the Clinical Practice Research Datalink (CPRD) using stringent case-definition criteria. Rates shown by ethnicity and year, 2012-2023, with 95% confidence intervals (95% CI). ..... 16

Supplementary Table 5. Crude mortality rates in people with systemic lupus erythematosus (SLE) and matched controls by age group, with 95% confidence intervals (95% CI). ..... 17

Supplementary Table 6. Number of individuals with systemic lupus erythematosus (SLE) at risk of all-cause mortality overall and by time since diagnosis, stratified by ethnicity. ..... 18

Supplementary Table 7. Hazard ratios for all-cause mortality within the systemic lupus erythematosus (SLE) cohort by ethnicity, subdividing into Black African and Black other ethnicities. ..... 19

Supplementary Table 8. Hazard ratios (HRs) for all-cause mortality using a stringent case-definition for systemic lupus erythematosus (SLE). ..... 20

Supplementary Table 9. Age and sex-standardised complication rates (ASCRs) in people with systemic lupus erythematosus (SLE) and matched controls, with 95% confidence intervals (95% CI). ..... 21

Supplementary Table 10. Number of individuals with pre-existing diagnoses excluded from age-standardised complication rate calculations. ..... 23

Supplementary Table 11. Hazard ratios for developing complications of interest in people with systemic lupus erythematosus (SLE) compared with matched controls.......................................... 24

Supplementary Table 12. Hazard ratios for developing complications of interest in males compared with females.......................................................................................................................... 25

Supplementary Table 13. Hazard ratios for developing complications of interest by age decile. .... 26

Supplementary Table 14. Solid and haematological cancer outcomes in people with systemic lupus erythematosus (SLE) and matched controls. ..................................................................... 27

Supplementary Table 15. Age and sex-standardised complication rates (ASCRs) per 1,000 person-years in people with systemic lupus erythematosus (SLE) by ethnicity, with 95% confidence intervals (95% CI). .......................................................................................................... 28

Supplementary Table 16. Hazard ratios for developing complications of interest in people with systemic lupus erythematosus (SLE) by ethnicity. ........................................................... 30

Supplementary Table 17. Hazard ratios for developing myocarditis/pericarditis, interstitial lung disease (ILD) or thrombosis in Black patients with systemic lupus erythematosus (SLE)................ 31

## Supplementary Figures

Supplementary Figure 1. Directed acyclic graphs (DAGs) for outcomes of interest.

Directed acyclic graphs illustrating assumed causal structures for: (a) all-cause mortality, (b) ischaemic heart disease (IHD), heart failure, stroke and transient ischaemic attacks (TIAs), chronic kidney disease (CKD), (c) diabetes, (d) thrombosis, (e) myocarditis and pericarditis, (f) interstitial lung disease (ILD), (g) solid cancers, (h) osteoporosis, (i) fractures, and (j) fibromyalgia. Abbreviations: SLE, systemic lupus erythematosus; BMI, body mass index.

a)

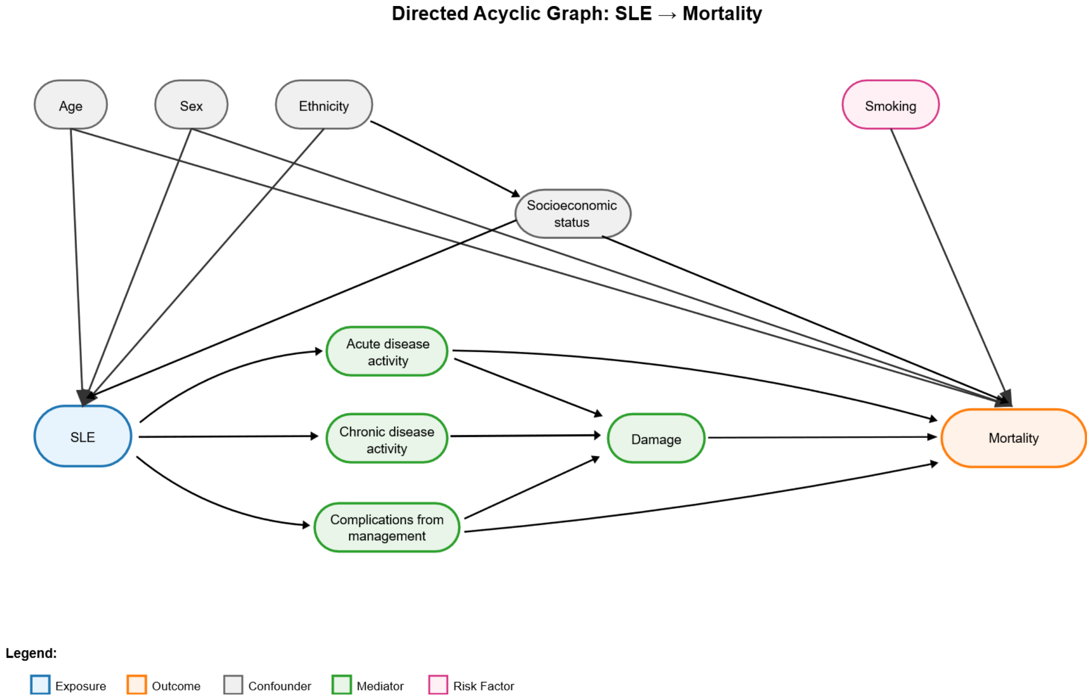

b)

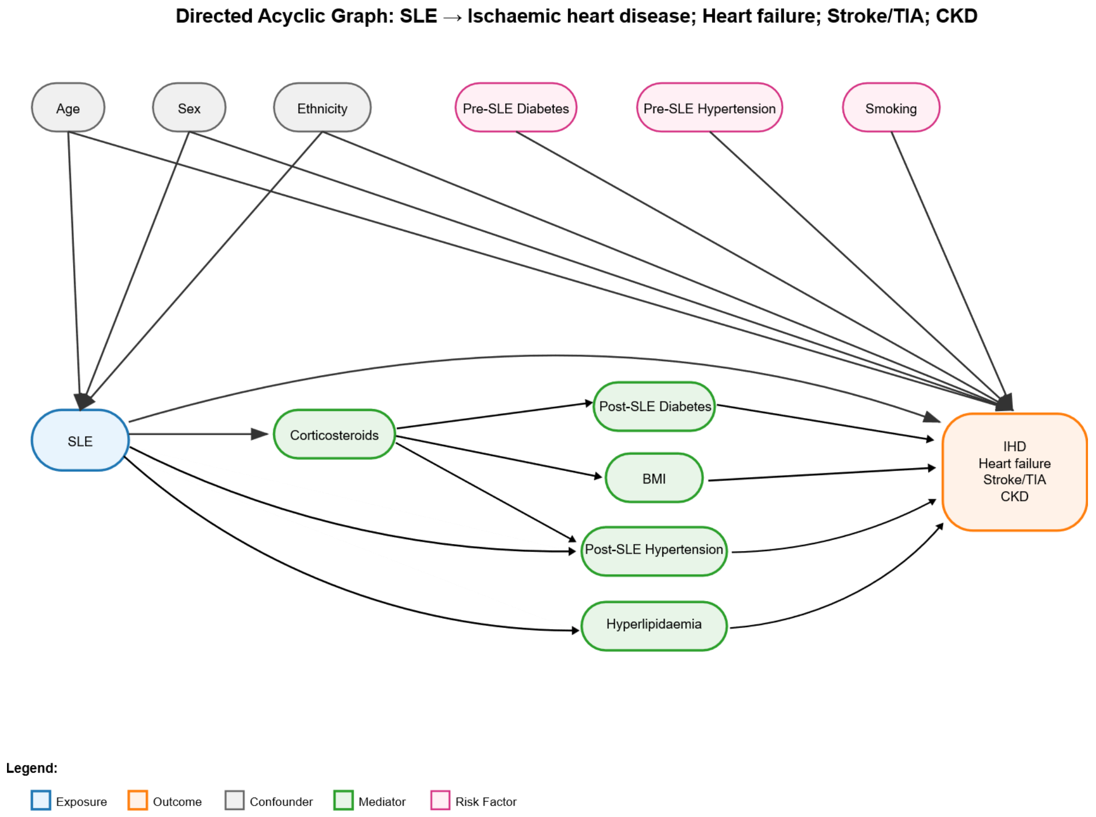
Directed Acyclic Graph: SLE → Ischaemic heart disease; Heart failure; Stroke/TIA; CKD
Age
Sex
Ethnicity
Pre-SLE Diabetes
Pre-SLE Hypertension
Smoking
SLE
Corticosteroids
Post-SLE Diabetes
BMI
Post-SLE Hypertension
Hyperlipidaemia
IHD
Heart failure
Stroke/TIA
CKD
Legend:
Exposure
Outcome
Confounder
Mediator
Risk Factor

c)

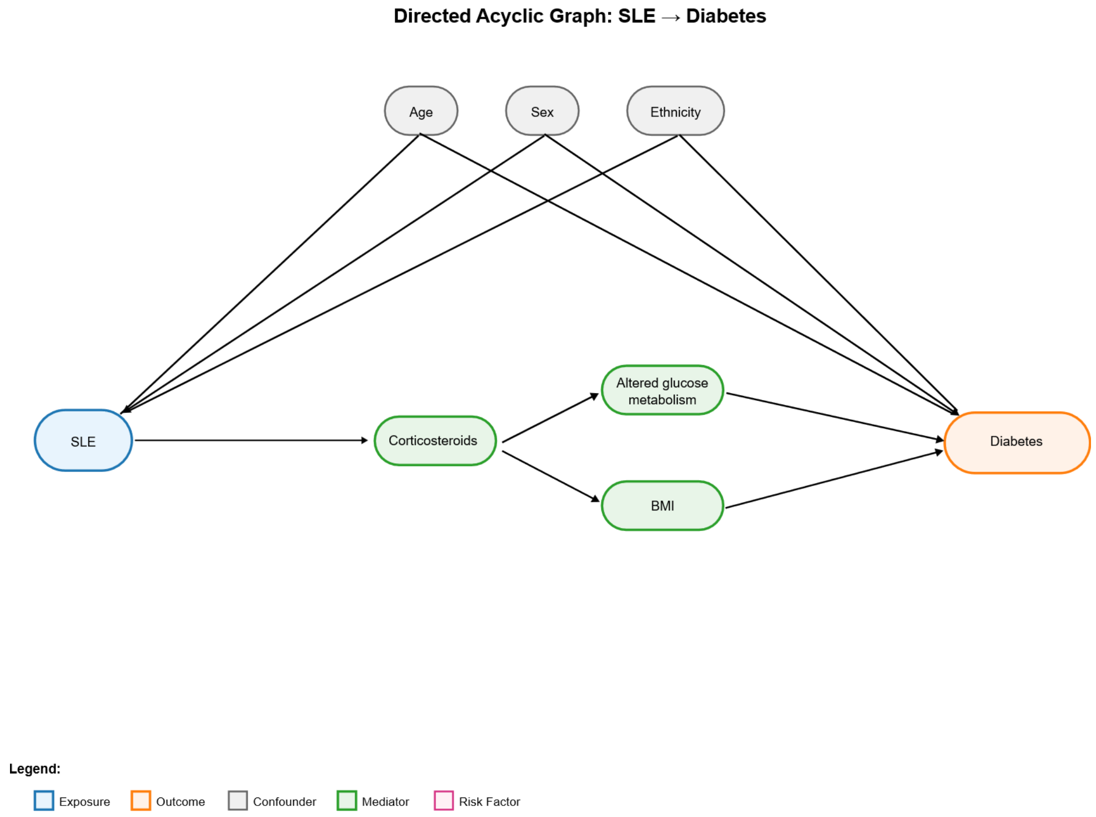
Directed Acyclic Graph: SLE → Diabetes
Age
Sex
Ethnicity
SLE
Corticosteroids
Altered glucose metabolism
BMI
Diabetes
Legend:
Exposure
Outcome
Confounder
Mediator
Risk Factor

d)

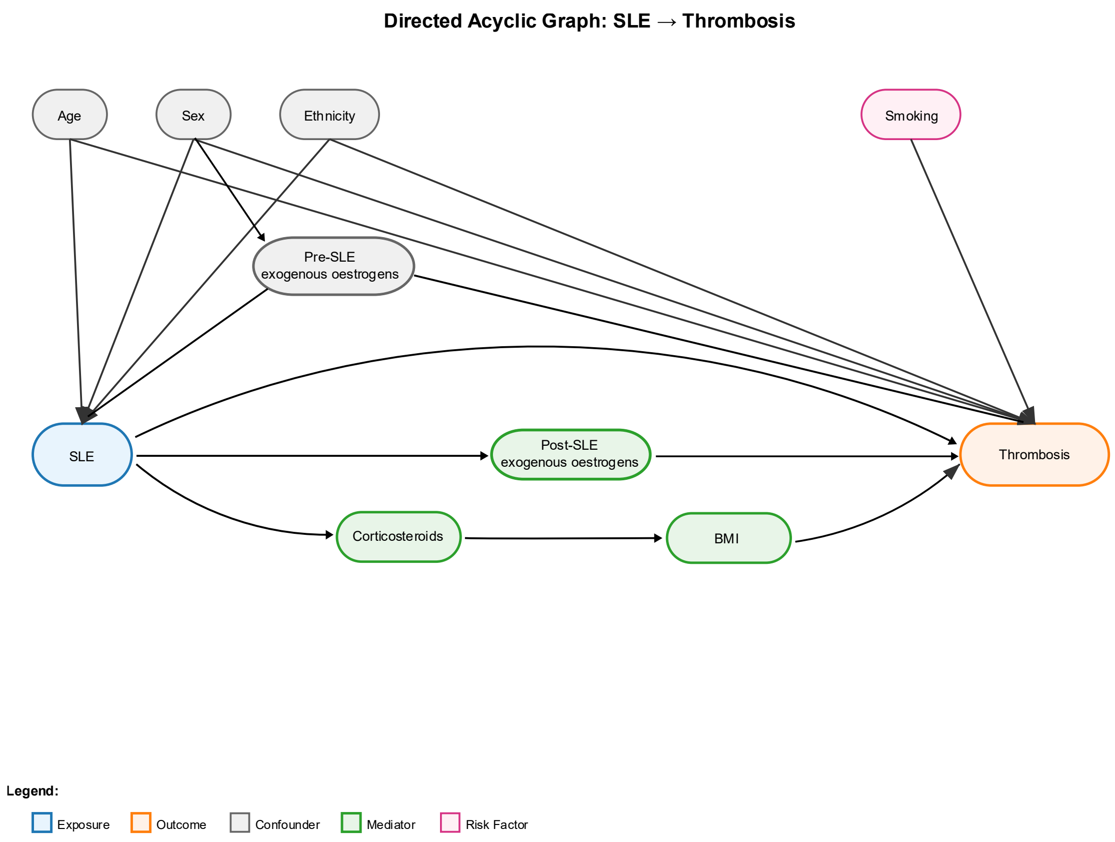
Directed Acyclic Graph: SLE → Thrombosis
Age
Sex
Ethnicity
Smoking
Pre-SLE
exogenous oestrogens
SLE
Post-SLE
exogenous oestrogens
Thrombosis
Corticosteroids
BMI
Legend:
Exposure
Outcome
Confounder
Mediator
Risk Factor

e)

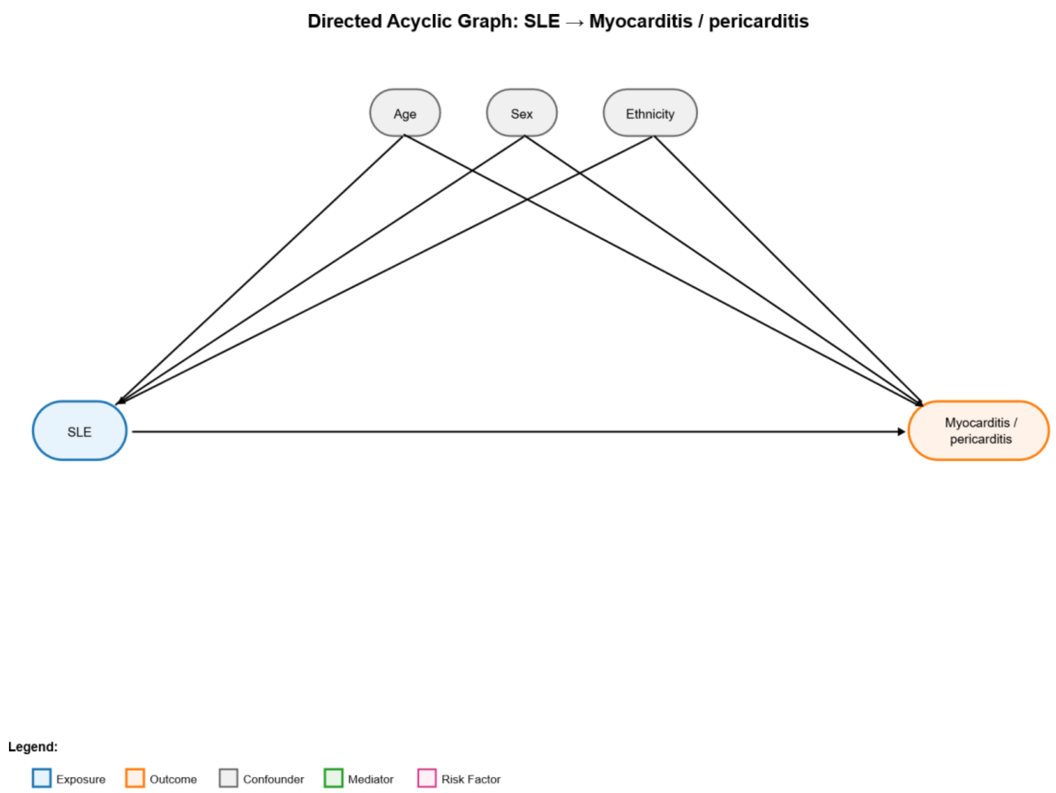
Directed Acyclic Graph: SLE → Myocarditis / pericarditis
Age
Sex
Ethnicity
SLE
Myocarditis /
pericarditis
Legend:
Exposure
Outcome
Confounder
Mediator
Risk Factor

f)

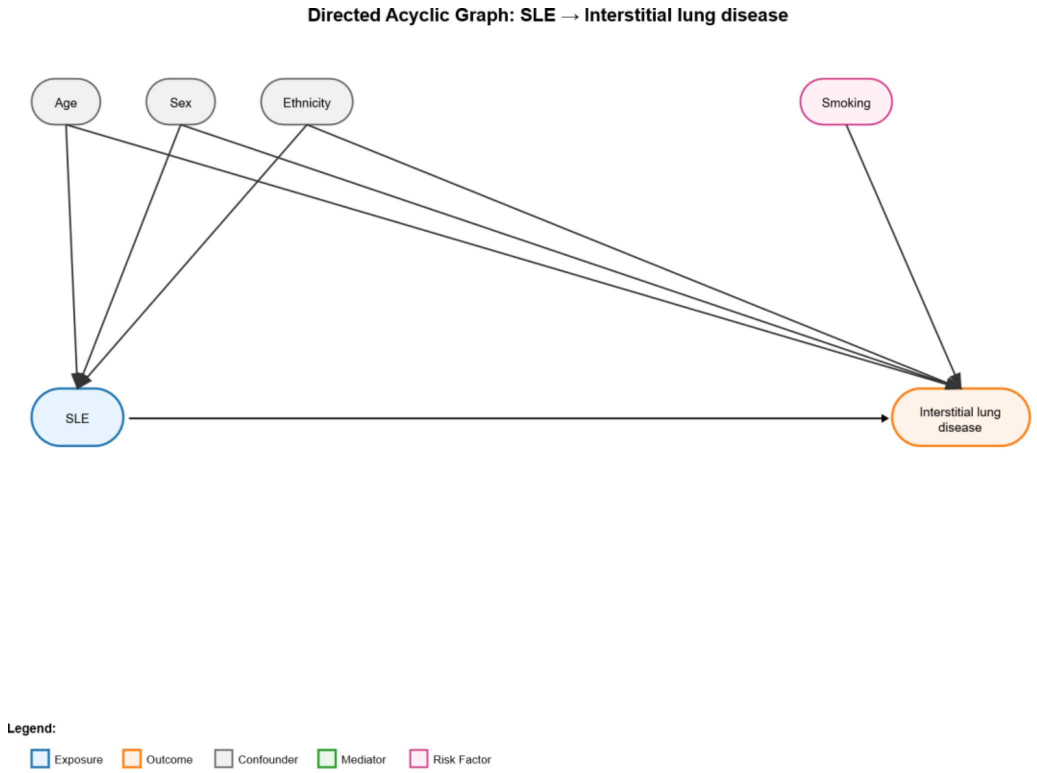
Directed Acyclic Graph: SLE → Interstitial lung disease
Age
Sex
Ethnicity
Smoking
SLE
Interstitial lung disease
Legend:
Exposure
Outcome
Confounder
Mediator
Risk Factor

g)

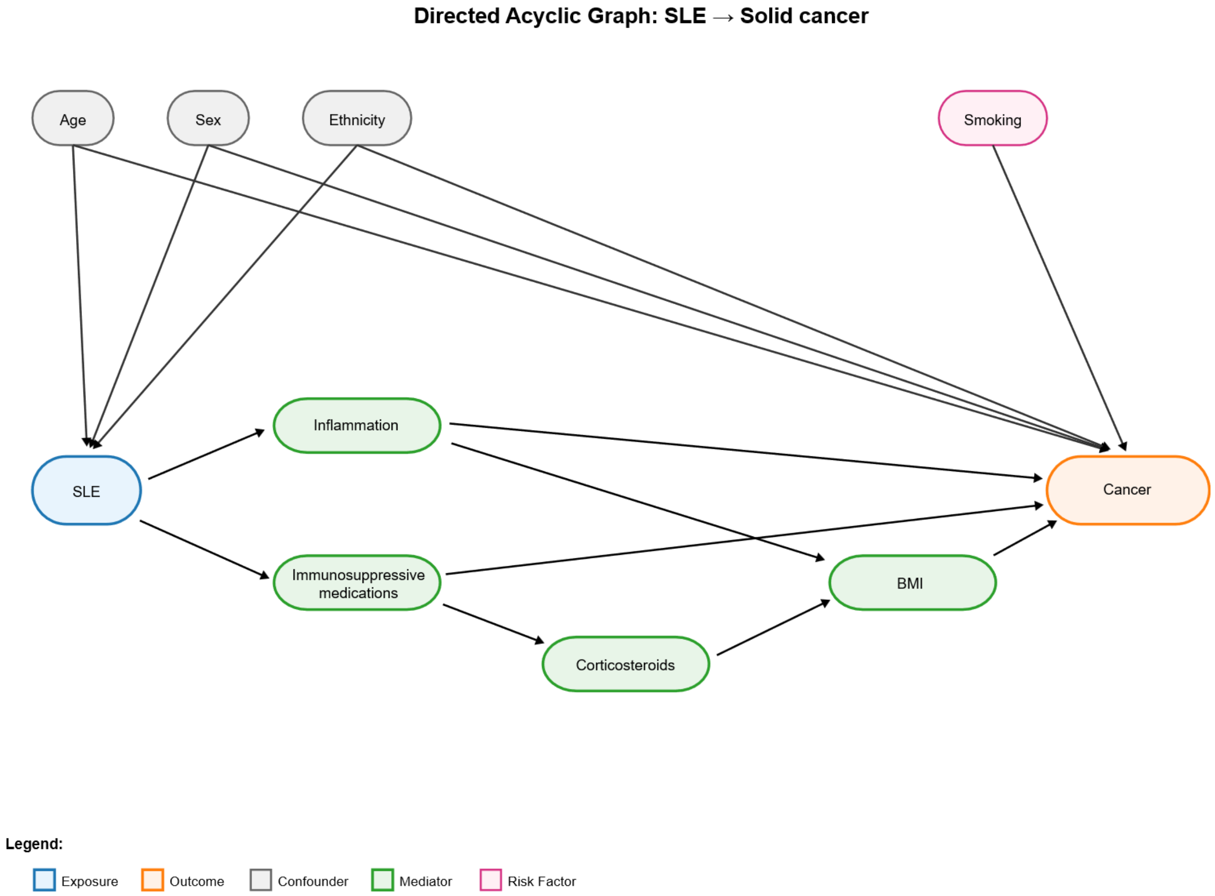
Directed Acyclic Graph: SLE → Solid cancer
Age
Sex
Ethnicity
Smoking
Inflammation
SLE
Cancer
Immunosuppressive medications
BMI
Corticosteroids
Legend:
Exposure
Outcome
Confounder
Mediator
Risk Factor

h)

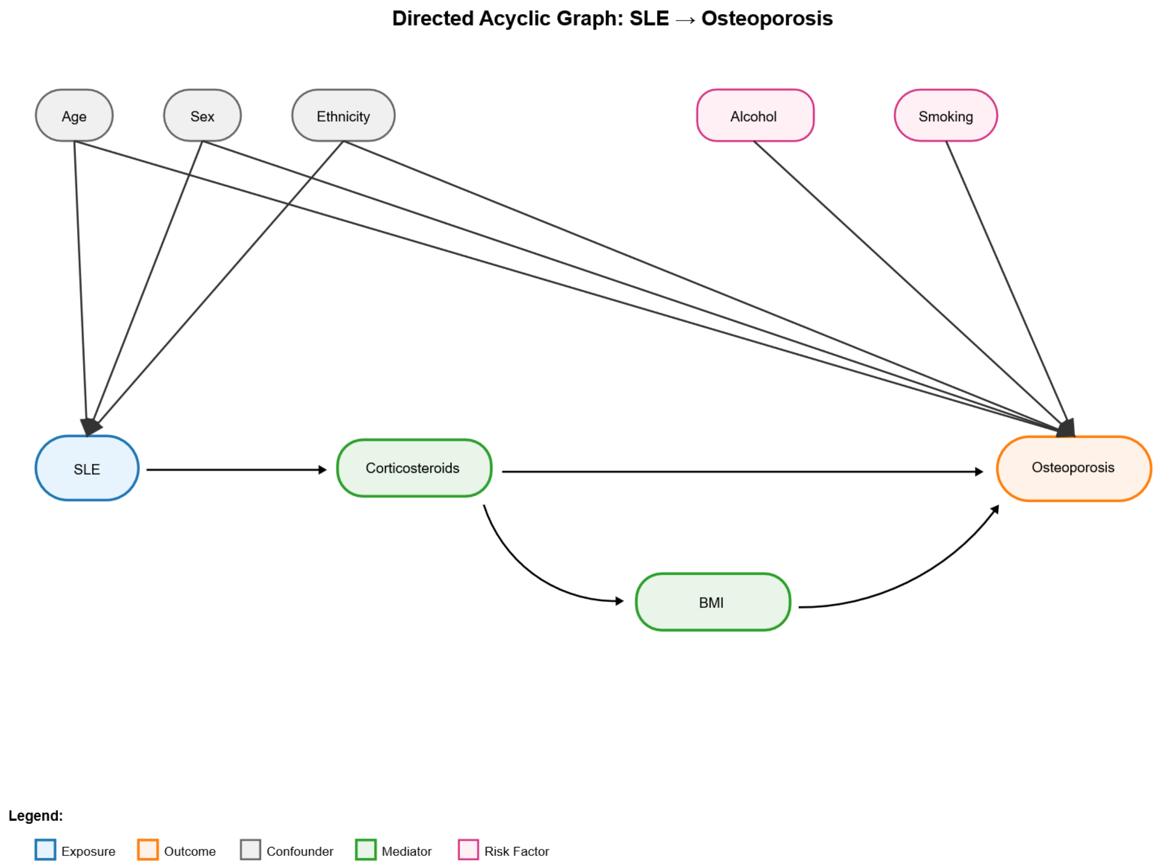
Directed Acyclic Graph: SLE → Osteoporosis
Age
Sex
Ethnicity
Alcohol
Smoking
SLE
Corticosteroids
Osteoporosis
BMI
Legend:
Exposure
Outcome
Confounder
Mediator
Risk Factor

i)

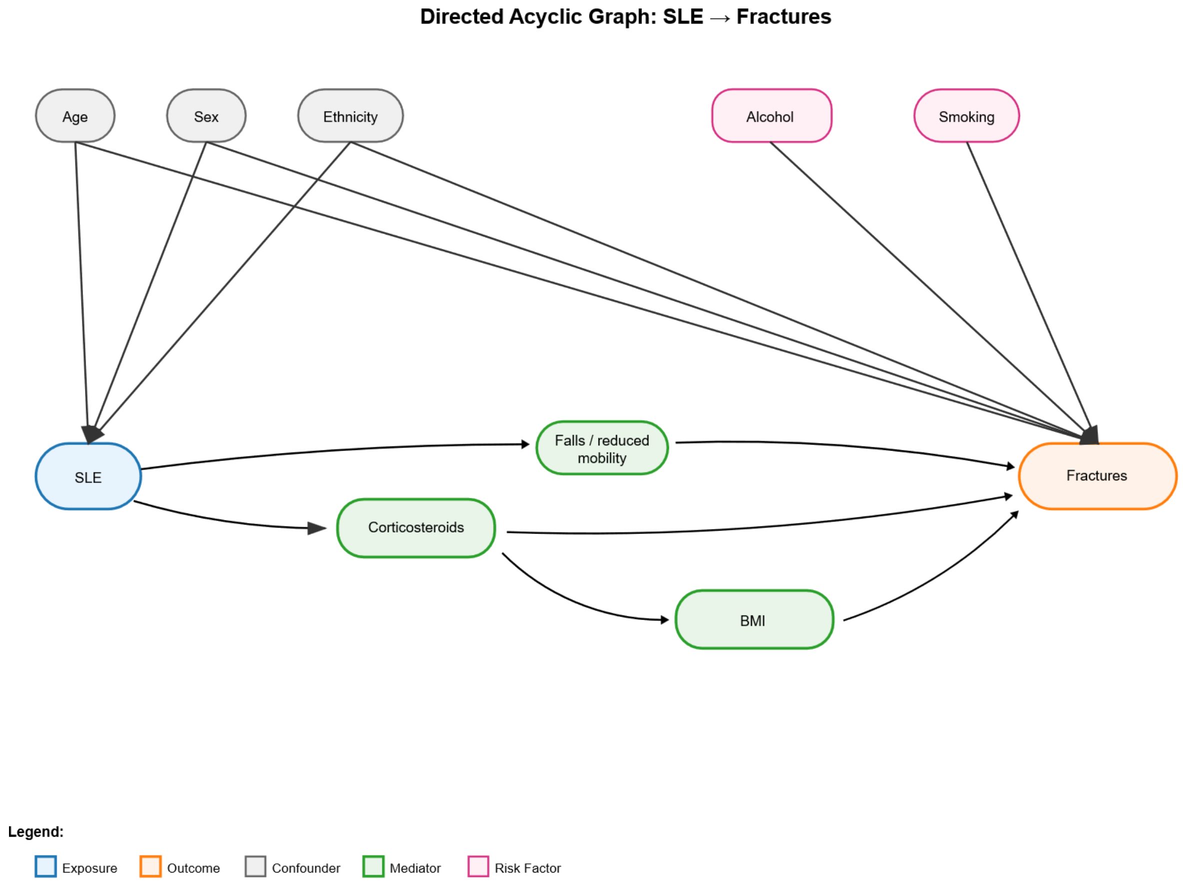
Directed Acyclic Graph: SLE → Fractures
Age
Sex
Ethnicity
Alcohol
Smoking
Falls / reduced mobility
SLE
Fractures
Corticosteroids
BMI
Legend:
Exposure
Outcome
Confounder
Mediator
Risk Factor

j)

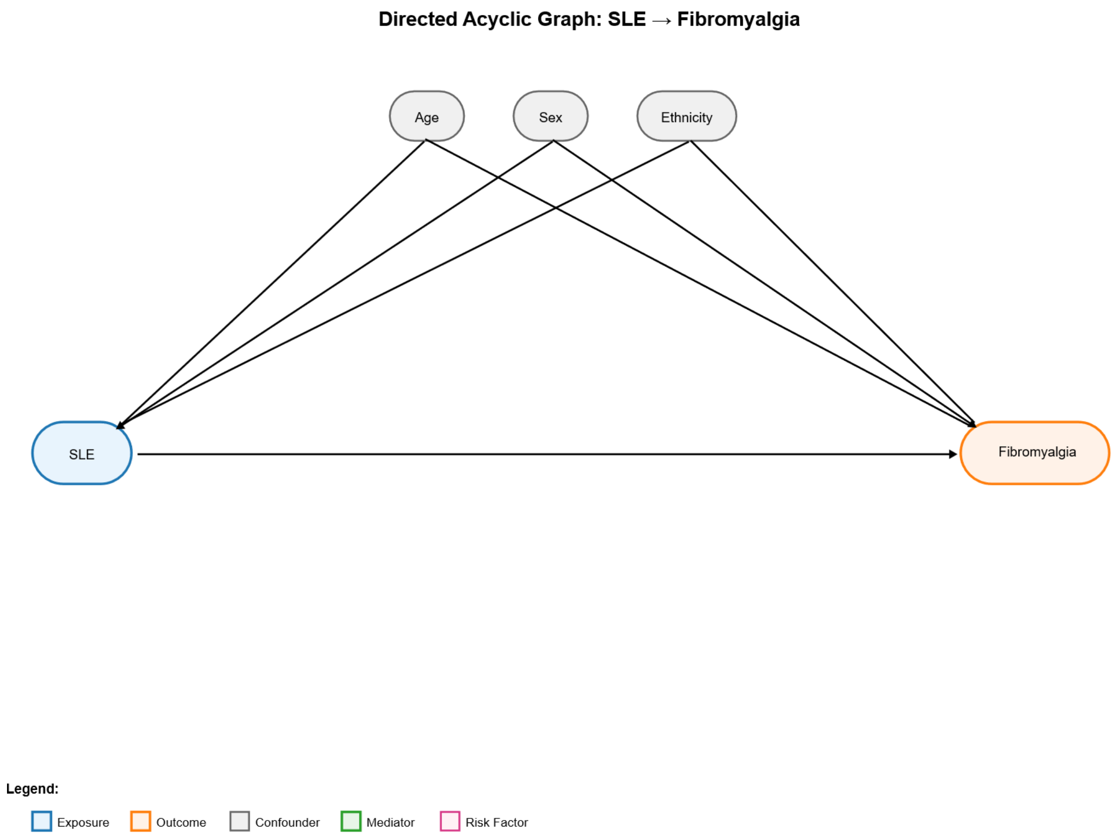
Directed Acyclic Graph: SLE → Fibromyalgia
Age
Sex
Ethnicity
SLE
Fibromyalgia
Legend:
Exposure
Outcome
Confounder
Mediator
Risk Factor

Supplementary Figure 2. Age and sex-standardised incidence rates of systemic lupus erythematosus (SLE) in the Clinical Practice Research Datalink (CPRD) using stringent case-definition criteria. Rates shown by ethnicity and year, 2012-2023.

Incidence rates are presented per 100,000 person-years with 95% confidence intervals (95% CI). Rates were age- and sex-standardised using direct standardisation against the 2013 European Standard Population.

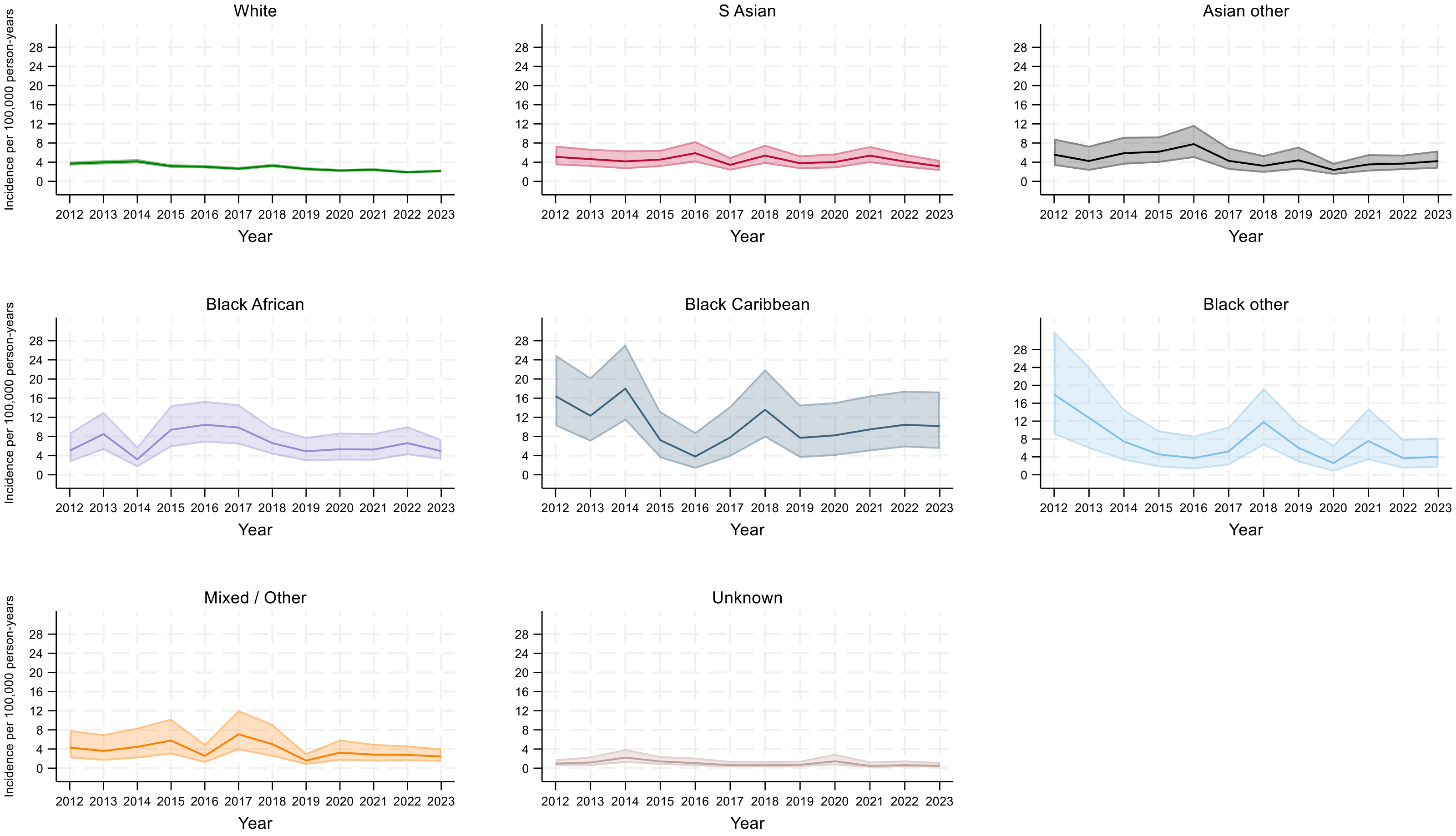

Supplementary Figure 3. Risk of death following diagnosis of systemic lupus erythematosus (SLE) over time.

Hazard functions were modelled using flexible parametric survival curves with restricted cubic splines. Shaded areas represent 95% confidence intervals. Models were adjusted for age, sex, and smoking status.

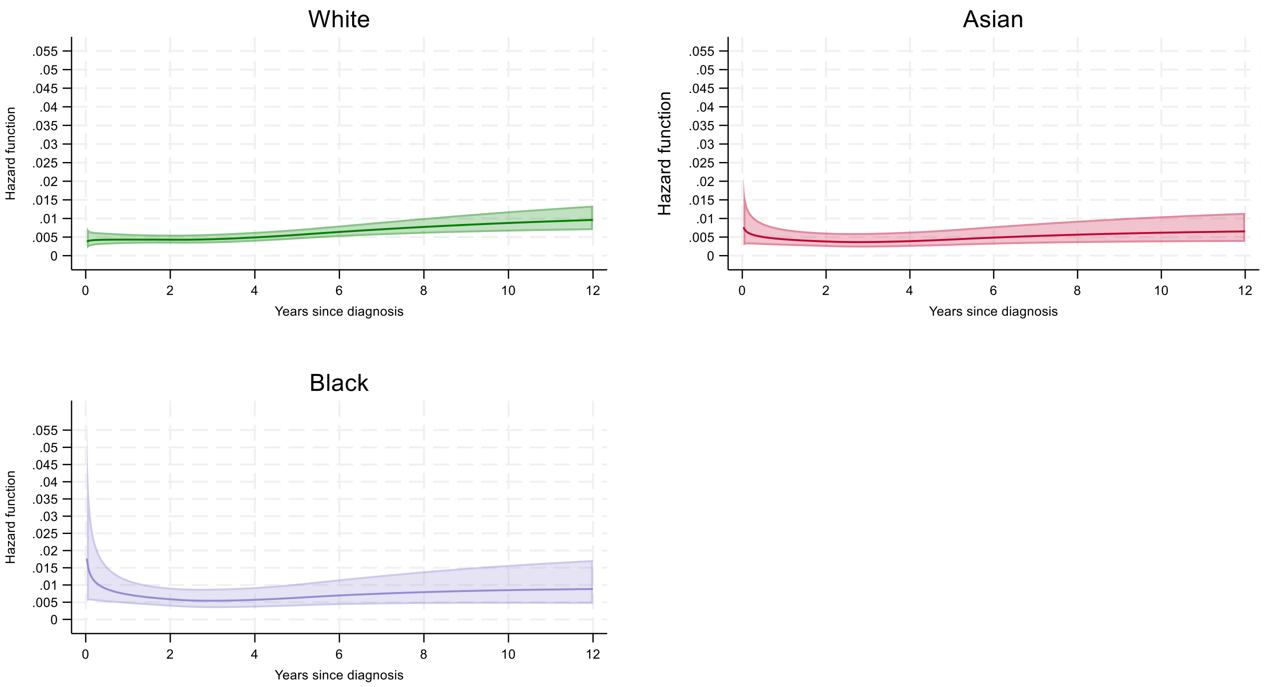

Supplementary Figure 4. Risk of developing complications of interest in men compared with women.

Estimates were obtained using Fine and Gray competing risk models. Hazard ratios represent the relative risk of developing complications in men compared with women and are shown with 95% confidence intervals represented as bars. All models were adjusted for ethnicity and accounted for age through matching. Additional covariates were selected based on directed acyclic graphs: * Prior diabetes, prior hypertension, smoking status. ‡ Smoking status, prior oestrogen use. † Smoking status. § Alcohol dependence, smoking status. Abbreviations: TIA, transient ischaemic attack; CKD, chronic kidney disease; ILD, interstitial lung disease.

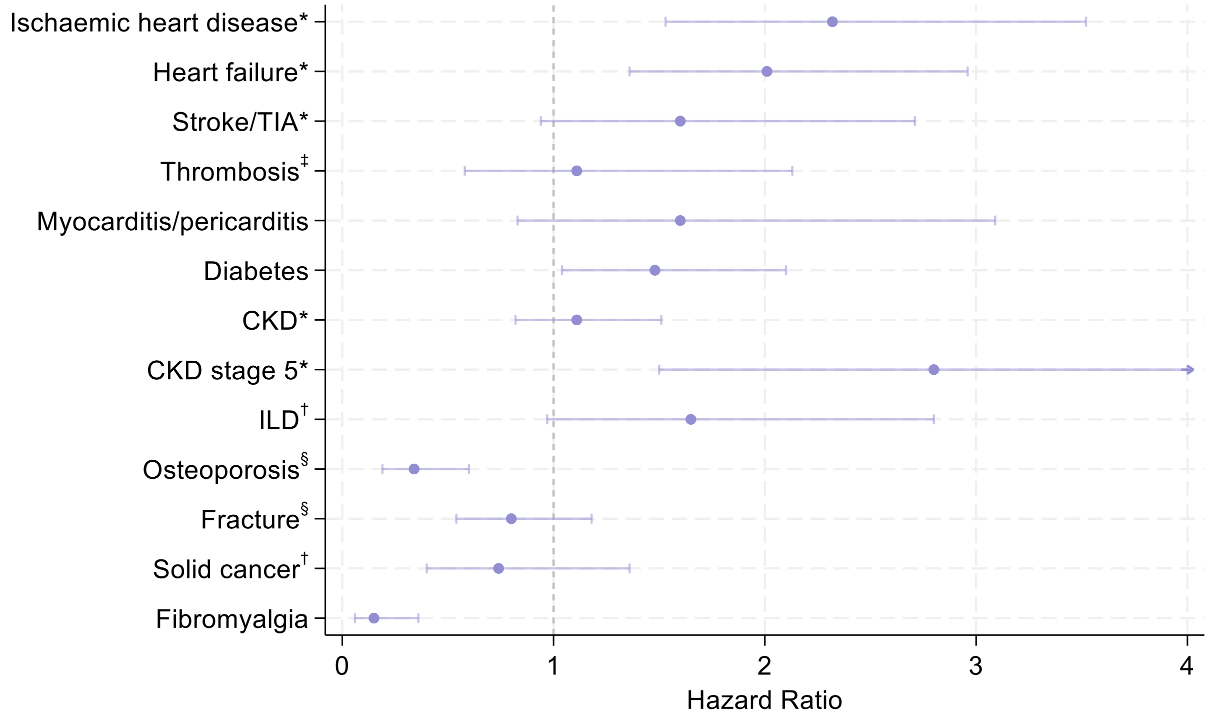

Supplementary Figure 5. Association between age and risk of developing complications of interest in people with systemic lupus erythematosus (SLE), with age modelled as a continuous variable in deciles.

Estimates were obtained using Fine and Gray competing risk models. Hazard ratios represent the relative change in risk per one-unit increase in age decile and are shown with 95% confidence intervals, represented as bars. All models were adjusted for ethnicity and accounted for age through matching. Additional covariates were selected based on directed acyclic graphs: * Prior diabetes, prior hypertension, smoking status. ‡ Smoking status, prior oestrogen use. † Smoking status. § Alcohol dependence, smoking status. Abbreviations: TIA, transient ischaemic attack; CKD, chronic kidney disease; ILD, interstitial lung disease.

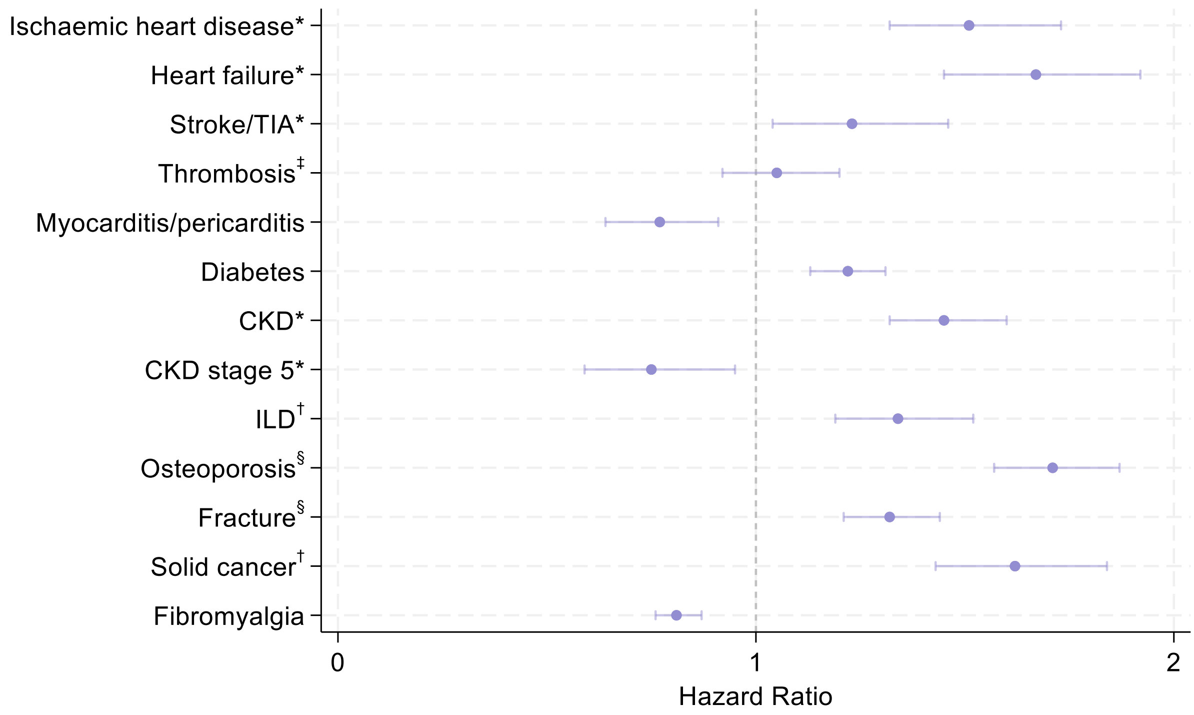

## Supplementary Tables

Supplementary Table 1. SNOMED-CT code lists used to define systemic lupus erythematosus (SLE), comorbidities, and complications of interest.

SNOMED-CT codes were used to identify diagnoses of SLE, baseline comorbidities, and incident complications within primary care records. Codes were selected based on clinical relevance and prior literature and applied consistently to cases and controls. Abbreviations: SNOMED-CT, Systematized Nomenclature of Medicine Clinical Terms.

Available at: https://doi.org/10.5281/zenodo.18171148

Supplementary Table 2. Baseline characteristics of patients with incident systemic lupus erythematosus (SLE) by ethnicity in the Clinical Practice Research Datalink (CPRD) from 2012 to 2023.

Baseline characteristics were assessed at the index date, defined as the date of SLE diagnosis. Data are presented as number (percentage) unless otherwise stated. Individuals with unknown ethnicity are not shown. Low counts <8 redacted (*). Abbreviations: IQR, interquartile range; IMD, index of multiple deprivation; TIA, transient ischaemic attack; CKD, chronic kidney disease; S Asian, South Asian.

| | White | S Asian | Asian other | Black African | Black Caribbean | Black other | Mixed / Other |
|---|---|---|---|---|---|---|---|
| | N=3,509 | N=423 | N=236 | N=253 | N=171 | N=107 | N=145 |
| **Median age at diagnosis (IQR)** | 50.0 (38.0,63.0) | 41.0 (33.0,52.0) | 40.0 (30.0,49.0) | 39.0 (29.0,50.0) | 45.0 (36.0,55.0) | 38.0 (29.0,48.0) | 37.0 (27.0,46.0) |
| **Female (%)** | 3,061 (87.2%) | 364 (86.1%) | 210 (89.0%) | 233 (92.1%) | 159 (93.0%) | 99 (92.5%) | 134 (92.4%) |
| **IMD quintile** | | | | | | | |
| 1 (Least deprived) | 631 (18.0%) | 42 (9.9%) | 14 (5.9%) | 14 (5.5%) | 12 (7.0%) | * | 19 (13.1%) |
| 2 | 656 (18.7%) | 34 (8.0%) | 27 (11.4%) | 12 (4.7%) | 10 (5.8%) | * | 15 (10.3%) |
| 3 | 587 (16.7%) | 76 (18.0%) | 54 (22.9%) | 26 (10.3%) | 27 (15.8%) | 20 (18.7%) | 21 (14.5%) |
| 4 | 548 (15.6%) | 77 (18.2%) | 61 (25.8%) | 77 (30.4%) | 40 (23.4%) | 39 (36.4%) | 26 (17.9%) |
| 5 (Most deprived) | 521 (14.8%) | 106 (25.1%) | 41 (17.4%) | 85 (33.6%) | 62 (36.3%) | 22 (20.6%) | 33 (22.8%) |
| Missing | 566 (16.1%) | 88 (20.8%) | 39 (16.5%) | 39 (15.4%) | 20 (11.7%) | 19 (17.8%) | 31 (21.4%) |
| **Smoking status** | | | | | | | |
| Non-Smoker | 1,423 (40.6%) | 329 (77.8%) | 183 (77.5%) | 203 (80.2%) | 93 (54.4%) | 75 (70.1%) | 85 (58.6%) |
| Ex-smoker | 1,348 (38.4%) | 51 (12.1%) | 34 (14.4%) | 26 (10.3%) | 40 (23.4%) | 15 (14.0%) | 32 (22.1%) |
| Current smoker | 738 (21.0%) | 43 (10.2%) | 19 (8.1%) | 24 (9.5%) | 38 (22.2%) | 17 (15.9%) | 28 (19.3%) |
| **Co-morbidities prior to SLE diagnosis** | | | | | | | |
| Diabetes mellitus | 257 (7.3%) | 51 (12.1%) | 14 (5.9%) | 29 (11.5%) | 13 (7.6%) | 8 (7.5%) | * |
| Hypertension | 845 (24.1%) | 80 (18.9%) | 35 (14.8%) | 60 (23.7%) | 48 (28.1%) | 16 (15.0%) | 12 (8.3%) |
| Ischaemic heart disease | 239 (6.8%) | 20 (4.7%) | 8 (3.4%) | * | * | * | * |
| Heart failure | 78 (2.2%) | * | * | * | * | * | * |
| Stroke / TIA | 149 (4.2%) | 12 (2.8%) | * | * | * | * | * |
| CKD stage 3+ | 355 (10.1%) | 18 (4.3%) | * | 19 (7.5%) | 13 (7.6%) | * | * |
| Interstitial lung disease | 49 (1.4%) | 8 (1.9%) | * | * | * | * | * |
| Solid cancers | 96 (2.7%) | * | * | * | * | * | * |
| Osteoporosis | 156 (4.4%) | 10 (2.4%) | * | * | * | * | * |
| Fibromyalgia | 244 (7.0%) | 18 (4.3%) | * | * | 8 (4.7%) | * | * |

Supplementary Table 3. Age and sex-standardised incidence rates (ASIRs) of systemic lupus erythematosus (SLE) in the Clinical Practice Research Datalink (CPRD) by ethnicity and year, 2012-2023, with 95% confidence intervals (95% CI).

| **Calendar year** | **White** | | **S Asian** | | **Asian other** | | **Black African** | | **Black Caribbean** | | **Black other** | | **Mixed / Other** | | **Unknown** | |
|---|---|---|---|---|---|---|---|---|---|---|---|---|---|---|---|---|
| | Crude incidence | Age standardised incidence | Crude incidence | Age standardised incidence | Crude incidence | Age standardised incidence | Crude incidence | Age standardised incidence | Crude incidence | Age standardised incidence | Crude incidence | Age standardised incidence | Crude incidence | Age standardised incidence | Crude incidence | Age standardised incidence |
| 2012 | 3.91 (3.49 - 4.36) | 4.23 (3.78 - 4.72) | 6.25 (4.28 - 8.83) | 6.46 (4.42 - 9.12) | 5.58 (3.31 - 8.82) | 5.77 (3.42 - 9.12) | 8.05 (4.69 - 12.89) | 6.44 (3.75 - 10.31) | 23.02 (14.75 - 34.25) | 18.66 (11.96 - 27.77) | 13.91 (6.94 - 24.89) | 17.96 (8.97 - 32.13) | 6.18 (3.08 - 11.05) | 11.04 (5.51 - 19.76) | 1.26 (0.63 - 2.26) | 1.23 (0.62 - 2.21) |
| 2013 | 4.03 (3.61 - 4.49) | 4.33 (3.88 - 4.82) | 7.03 (4.95 - 9.70) | 7.60 (5.35 - 10.48) | 4.44 (2.48 - 7.32) | 4.63 (2.59 - 7.63) | 9.81 (6.07 - 15.00) | 8.83 (5.46 - 13.49) | 15.19 (8.68 - 24.66) | 13.11 (7.50 - 21.30) | 12.22 (5.86 - 22.47) | 13.39 (6.42 - 24.62) | 4.33 (1.87 - 8.52) | 3.60 (1.55 - 7.09) | 0.99 (0.43 - 1.96) | 1.45 (0.63 - 2.86) |
| 2014 | 4.29 (3.86 - 4.75) | 4.54 (4.09 - 5.04) | 4.22 (2.67 - 6.33) | 4.56 (2.89 - 6.84) | 5.11 (3.03 - 8.08) | 5.88 (3.49 - 9.30) | 5.44 (2.81 - 9.49) | 4.98 (2.57 - 8.70) | 21.35 (13.53 - 32.03) | 18.75 (11.89 - 28.13) | 9.30 (4.01 - 18.32) | 7.46 (3.22 - 14.69) | 5.12 (2.46 - 9.42) | 4.94 (2.37 - 9.09) | 1.69 (0.90 - 2.89) | 2.41 (1.28 - 4.12) |
| 2015 | 3.57 (3.19 - 3.99) | 3.66 (3.27 - 4.09) | 5.97 (4.13 - 8.34) | 5.58 (3.87 - 7.80) | 6.52 (4.17 - 9.69) | 6.54 (4.19 - 9.74) | 10.00 (6.34 - 15.01) | 10.52 (6.67 - 15.79) | 9.00 (4.32 - 16.55) | 7.23 (3.47 - 13.29) | 8.80 (3.80 - 17.33) | 6.22 (2.69 - 12.26) | 6.72 (3.67 - 11.28) | 7.18 (3.93 - 12.05) | 1.47 (0.73 - 2.63) | 1.41 (0.70 - 2.52) |
| 2016 | 3.47 (3.10 - 3.88) | 3.53 (3.15 - 3.94) | 5.02 (3.39 - 7.16) | 5.88 (3.97 - 8.39) | 5.87 (3.72 - 8.80) | 7.95 (5.04 - 11.92) | 12.42 (8.38 - 17.74) | 13.57 (9.16 - 19.38) | 5.22 (1.91 - 11.36) | 4.53 (1.66 - 9.85) | 7.26 (2.92 - 14.95) | 7.08 (2.85 - 14.59) | 4.00 (1.83 - 7.59) | 3.24 (1.48 - 6.15) | 1.08 (0.47 - 2.13) | 1.14 (0.49 - 2.24) |
| 2017 | 2.81 (2.48 - 3.17) | 2.82 (2.49 - 3.19) | 5.59 (3.89 - 7.77) | 4.69 (3.26 - 6.52) | 3.85 (2.20 - 6.25) | 4.44 (2.54 - 7.21) | 9.88 (6.40 - 14.59) | 11.75 (7.60 - 17.35) | 9.32 (4.65 - 16.67) | 8.42 (4.20 - 15.06) | 6.88 (2.77 - 14.18) | 5.22 (2.10 - 10.76) | 5.76 (3.15 - 9.66) | 7.26 (3.97 - 12.17) | 1.09 (0.47 - 2.15) | 1.16 (0.50 - 2.29) |
| 2018 | 3.70 (3.32 - 4.10) | 3.78 (3.40 - 4.20) | 5.95 (4.23 - 8.13) | 6.44 (4.58 - 8.81) | 3.85 (2.24 - 6.16) | 3.66 (2.13 - 5.87) | 9.82 (6.41 - 14.38) | 7.25 (4.74 - 10.62) | 14.09 (8.21 - 22.55) | 14.44 (8.41 - 23.12) | 14.94 (8.54 - 24.27) | 12.50 (7.15 - 20.31) | 4.20 (2.10 - 7.51) | 5.19 (2.59 - 9.29) | 0.82 (0.30 - 1.77) | 0.95 (0.35 - 2.06) |
| 2019 | 2.90 (2.57 - 3.26) | 2.96 (2.62 - 3.33) | 5.15 (3.59 - 7.17) | 4.83 (3.36 - 6.72) | 3.65 (2.13 - 5.85) | 4.92 (2.87 - 7.89) | 6.20 (3.61 - 9.92) | 4.91 (2.86 - 7.86) | 8.37 (4.02 - 15.40) | 8.68 (4.16 - 15.96) | 8.97 (4.30 - 16.49) | 6.50 (3.12 - 11.96) | 3.22 (1.47 - 6.11) | 1.94 (0.89 - 3.68) | 0.95 (0.38 - 1.96) | 0.86 (0.35 - 1.77) |
| 2020 | 2.59 (2.28 - 2.93) | 2.62 (2.31 - 2.97) | 4.47 (3.06 - 6.31) | 4.57 (3.13 - 6.45) | 3.26 (1.86 - 5.29) | 2.37 (1.36 - 3.86) | 5.90 (3.44 - 9.44) | 5.86 (3.41 - 9.38) | 9.02 (4.50 - 16.13) | 9.12 (4.55 - 16.32) | 5.11 (1.88 - 11.13) | 4.07 (1.49 - 8.85) | 3.33 (1.60 - 6.13) | 3.24 (1.56 - 5.96) | 0.95 (0.38 - 1.96) | 1.44 (0.58 - 2.98) |
| 2021 | 2.64 (2.33 - 2.98) | 2.68 (2.36 - 3.02) | 5.83 (4.24 - 7.83) | 6.20 (4.50 - 8.32) | 3.29 (1.92 - 5.27) | 3.54 (2.06 - 5.67) | 5.94 (3.52 - 9.39) | 5.99 (3.55 - 9.46) | 10.53 (5.61 - 18.00) | 10.37 (5.52 - 17.74) | 6.44 (2.78 - 12.70) | 7.57 (3.27 - 14.91) | 3.67 (1.89 - 6.40) | 3.03 (1.56 - 5.29) | 0.42 (0.09 - 1.23) | 0.48 (0.10 - 1.40) |
| 2022 | 2.21 (1.93 - 2.53) | 2.23 (1.95 - 2.55) | 5.35 (3.89 - 7.19) | 5.02 (3.65 - 6.74) | 4.38 (2.83 - 6.46) | 3.92 (2.53 - 5.78) | 6.79 (4.26 - 10.28) | 6.92 (4.34 - 10.47) | 12.92 (7.39 - 20.98) | 11.88 (6.79 - 19.30) | 5.30 (2.13 - 10.92) | 4.09 (1.64 - 8.42) | 5.00 (2.96 - 7.91) | 3.59 (2.13 - 5.67) | 0.59 (0.16 - 1.52) | 0.63 (0.17 - 1.62) |
| 2023 | 2.37 (2.08 - 2.70) | 2.42 (2.12 - 2.76) | 4.14 (2.93 - 5.68) | 3.80 (2.69 - 5.22) | 4.79 (3.23 - 6.83) | 5.67 (3.83 - 8.10) | 6.93 (4.48 - 10.23) | 5.44 (3.52 - 8.03) | 11.14 (6.09 - 18.70) | 11.07 (6.05 - 18.58) | 6.24 (2.86 - 11.85) | 4.95 (2.26 - 9.39) | 4.80 (2.89 - 7.49) | 3.62 (2.18 - 5.65) | 1.05 (0.42 - 2.16) | 1.18 (0.47 - 2.43) |

Supplementary Table 4. Age and sex-standardised incidence rates (ASIRs) of systemic lupus erythematosus (SLE) in the Clinical Practice Research Datalink (CPRD) using stringent case-definition criteria. Rates shown by ethnicity and year, 2012-2023, with 95% confidence intervals (95% CI).

| **Calendar year** | **White** | | **S Asian** | | **Asian other** | | **Black African** | | **Black Caribbean** | | **Black other** | | **Mixed / Other** | | **Unknown** | |
|---|---|---|---|---|---|---|---|---|---|---|---|---|---|---|---|---|
| | Crude incidence | Age standardised incidence | Crude incidence | Age standardised incidence | Crude incidence | Age standardised incidence | Crude incidence | Age standardised incidence | Crude incidence | Age standardised incidence | Crude incidence | Age standardised incidence | Crude incidence | Age standardised incidence | Crude incidence | Age standardised incidence |
| 2012 | 3.48 (3.09 - 3.91) | 3.70 (3.28 - 4.15) | 5.28 (3.48 - 7.68) | 5.11 (3.37 - 7.44) | 5.27 (3.07 - 8.44) | 5.58 (3.25 - 8.93) | 5.68 (2.94 - 9.93) | 5.03 (2.60 - 8.78) | 20.14 (12.47 - 30.79) | 16.43 (10.17 - 25.11) | 13.91 (6.94 - 24.89) | 17.96 (8.97 - 32.13) | 5.62 (2.69 - 10.33) | 4.32 (2.07 - 7.94) | 1.03 (0.47 - 1.96) | 0.96 (0.44 - 1.83) |
| 2013 | 3.72 (3.32 - 4.16) | 3.96 (3.53 - 4.43) | 4.94 (3.23 - 7.24) | 4.64 (3.03 - 6.79) | 3.55 (1.83 - 6.20) | 4.25 (2.20 - 7.43) | 9.34 (5.71 - 14.43) | 8.50 (5.19 - 13.12) | 14.24 (7.97 - 23.48) | 12.33 (6.90 - 20.33) | 11.00 (5.03 - 20.87) | 12.73 (5.82 - 24.17) | 4.33 (1.87 - 8.52) | 3.60 (1.55 - 7.09) | 0.87 (0.35 - 1.79) | 1.18 (0.48 - 2.44) |
| 2014 | 3.97 (3.56 - 4.42) | 4.18 (3.75 - 4.65) | 3.67 (2.24 - 5.66) | 4.18 (2.56 - 6.46) | 5.11 (3.03 - 8.08) | 5.88 (3.49 - 9.30) | 4.53 (2.17 - 8.33) | 3.21 (1.54 - 5.91) | 20.42 (12.80 - 30.91) | 18.00 (11.28 - 27.25) | 9.30 (4.01 - 18.32) | 7.46 (3.22 - 14.69) | 4.61 (2.11 - 8.75) | 4.45 (2.04 - 8.46) | 1.43 (0.71 - 2.56) | 2.22 (1.11 - 3.97) |
| 2015 | 3.13 (2.77 - 3.52) | 3.18 (2.81 - 3.58) | 4.91 (3.26 - 7.10) | 4.54 (3.02 - 6.56) | 5.97 (3.74 - 9.04) | 6.19 (3.88 - 9.37) | 8.70 (5.31 - 13.44) | 9.41 (5.75 - 14.54) | 9.00 (4.32 - 16.55) | 7.23 (3.47 - 13.29) | 6.60 (2.42 - 14.36) | 4.56 (1.67 - 9.93) | 5.28 (2.64 - 9.45) | 5.77 (2.88 - 10.33) | 1.47 (0.73 - 2.63) | 1.41 (0.70 - 2.52) |
| 2016 | 3.02 (2.67 - 3.40) | 3.04 (2.69 - 3.43) | 5.02 (3.39 - 7.16) | 5.88 (3.97 - 8.39) | 5.61 (3.52 - 8.50) | 7.78 (4.87 - 11.77) | 10.35 (6.70 - 15.28) | 10.44 (6.76 - 15.41) | 4.35 (1.41 - 10.15) | 3.82 (1.24 - 8.91) | 5.18 (1.68 - 12.10) | 3.74 (1.21 - 8.73) | 3.55 (1.53 - 7.00) | 2.57 (1.11 - 5.06) | 0.95 (0.38 - 1.95) | 1.07 (0.43 - 2.20) |
| 2017 | 2.64 (2.32 - 2.99) | 2.65 (2.33 - 3.00) | 4.15 (2.71 - 6.08) | 3.45 (2.25 - 5.05) | 3.61 (2.02 - 5.95) | 4.28 (2.39 - 7.06) | 9.49 (6.08 - 14.12) | 9.88 (6.33 - 14.69) | 8.47 (4.06 - 15.58) | 7.80 (3.74 - 14.35) | 6.88 (2.77 - 14.18) | 5.22 (2.10 - 10.76) | 5.35 (2.85 - 9.14) | 7.08 (3.77 - 12.10) | 0.68 (0.22 - 1.59) | 0.64 (0.21 - 1.50) |
| 2018 | 3.25 (2.89 - 3.63) | 3.29 (2.93 - 3.67) | 4.73 (3.21 - 6.71) | 5.38 (3.66 - 7.64) | 3.17 (1.73 - 5.31) | 3.25 (1.78 - 5.45) | 9.06 (5.81 - 13.48) | 6.61 (4.23 - 9.83) | 13.26 (7.58 - 21.53) | 13.58 (7.76 - 22.06) | 14.01 (7.84 - 23.11) | 11.78 (6.59 - 19.43) | 3.82 (1.83 - 7.02) | 5.02 (2.41 - 9.24) | 0.68 (0.22 - 1.59) | 0.63 (0.21 - 1.47) |
| 2019 | 2.53 (2.22 - 2.87) | 2.57 (2.26 - 2.92) | 4.42 (2.98 - 6.31) | 3.79 (2.55 - 5.40) | 3.22 (1.80 - 5.32) | 4.40 (2.46 - 7.26) | 6.20 (3.61 - 9.92) | 4.91 (2.86 - 7.86) | 7.54 (3.45 - 14.31) | 7.71 (3.53 - 14.64) | 8.07 (3.69 - 15.32) | 6.01 (2.75 - 11.41) | 2.86 (1.24 - 5.64) | 1.59 (0.69 - 3.14) | 0.81 (0.30 - 1.77) | 0.71 (0.26 - 1.55) |
| 2020 | 2.24 (1.96 - 2.56) | 2.26 (1.97 - 2.58) | 4.05 (2.71 - 5.82) | 4.05 (2.71 - 5.81) | 3.26 (1.86 - 5.29) | 2.37 (1.36 - 3.86) | 5.20 (2.91 - 8.58) | 5.34 (2.99 - 8.80) | 8.20 (3.93 - 15.08) | 8.24 (3.95 - 15.15) | 3.41 (0.93 - 8.73) | 2.61 (0.71 - 6.68) | 3.33 (1.60 - 6.13) | 3.24 (1.56 - 5.96) | 0.95 (0.38 - 1.96) | 1.44 (0.58 - 2.98) |
| 2021 | 2.39 (2.09 - 2.71) | 2.41 (2.11 - 2.74) | 5.17 (3.68 - 7.07) | 5.38 (3.82 - 7.35) | 3.29 (1.92 - 5.27) | 3.54 (2.06 - 5.67) | 4.95 (2.77 - 8.17) | 5.26 (2.95 - 8.68) | 9.72 (5.02 - 16.97) | 9.48 (4.90 - 16.55) | 6.44 (2.78 - 12.70) | 7.57 (3.27 - 14.91) | 3.36 (1.68 - 6.01) | 2.83 (1.41 - 5.07) | 0.42 (0.09 - 1.23) | 0.48 (0.10 - 1.40) |
| 2022 | 1.89 (1.62 - 2.18) | 1.91 (1.64 - 2.20) | 4.38 (3.07 - 6.07) | 4.14 (2.90 - 5.73) | 4.03 (2.55 - 6.04) | 3.71 (2.35 - 5.56) | 6.48 (4.01 - 9.91) | 6.62 (4.10 - 10.12) | 11.31 (6.18 - 18.97) | 10.45 (5.71 - 17.53) | 4.54 (1.67 - 9.89) | 3.68 (1.35 - 8.01) | 3.61 (1.92 - 6.18) | 2.77 (1.48 - 4.74) | 0.59 (0.16 - 1.52) | 0.63 (0.17 - 1.62) |
| 2023 | 2.11 (1.83 - 2.41) | 2.15 (1.87 - 2.47) | 3.48 (2.38 - 4.92) | 3.15 (2.16 - 4.45) | 3.51 (2.20 - 5.32) | 4.24 (2.66 - 6.42) | 6.38 (4.04 - 9.57) | 4.95 (3.14 - 7.42) | 10.35 (5.51 - 17.69) | 10.16 (5.41 - 17.38) | 4.86 (1.95 - 10.01) | 4.03 (1.62 - 8.31) | 3.54 (1.93 - 5.93) | 2.45 (1.34 - 4.11) | 0.60 (0.16 - 1.54) | 0.50 (0.14 - 1.27) |

Supplementary Table 5. Crude mortality rates in people with systemic lupus erythematosus (SLE) and matched controls by age group, with 95% confidence intervals (95% CI).

Crude mortality rates are presented per 1,000 person-years and stratified by age group. Mortality rates are shown separately for people with SLE and matched control participants. Data redacted for statistical disclosure control (*).

| Age group | SLE deaths (N) | SLE mortality rate | Control deaths (N) | Control mortality rate | Mortality rate ratio |
|---|---|---|---|---|---|
| 18-39 | 13 | 2.26 (1.20 - 3.87) | 9 | 0.43 (0.20 - 0.82) | 5.22 (2.23 - 12.21) |
| 40-49 | 18 | 3.37 (2.00 - 5.33) | 21 | 1.04 (0.65 - 1.60) | 3.23 (1.72 - 6.06) |
| 50-59 | 40 | 6.74 (4.82 - 9.18) | 63 | 2.71 (2.08 - 3.46) | 2.49 (1.68 - 3.70) |
| 60-69 | 80 | 19.91 (15.79 - 24.78) | 117 | 7.15 (5.91 - 8.57) | 2.79 (2.10 - 3.70) |
| 70-79 | 124 | 45.60 (37.92 - 54.36) | 238 | 20.33 (17.83 - 23.09) | 2.24 (1.80 - 2.79) |
| 80+ | 110 | 96.10 (78.99 - 115.83) | 303 | 59.80 (53.25 - 66.93) | 1.61 (1.29 - 2.00) |

Supplementary Table 6. Number of individuals with systemic lupus erythematosus (SLE) at risk of all-cause mortality overall and by time since diagnosis, stratified by ethnicity.

Number of individuals (N) at risk of all-cause mortality are shown overall and within time-from-diagnosis intervals (0-1 year, 1-5 years, and ≥5 years). Numbers shown for three largest ethnic groups: White, Asian and Black. Smaller ethnic categories (e.g. Mixed/Other, Unknown) are not shown due to small numbers.

| | **Overall (N)** | **0-1 year from diagnosis (n)** | **1-5 years from diagnosis (n)** | **≥5 years from diagnosis (n)** |
|---|---|---|---|---|
| White | 3507 | 3507 | 3076 | 1734 |
| Asian | 659 | 659 | 553 | 289 |
| Black | 531 | 531 | 438 | 241 |

Supplementary Table 7. Hazard ratios for all-cause mortality within the systemic lupus erythematosus (SLE) cohort by ethnicity, subdividing into Black African and Black other ethnicities.

Hazard ratios (HRs) with 95% confidence intervals (95% CI) were estimated using flexible parametric survival models within the SLE cohort. White ethnicity served as the reference group. Models were adjusted for age, sex, and smoking status. [a] Black overall HR derived from the primary model which included White, Asian and Black ethnicity categories (see Table 3). All other estimates in this table are from the sensitivity model with subdivided Black ethnicity categories.

| | **Overall HR (95% CIs)** |
|---|---|
| White | Reference |
| Asian | 0.96 (0.60 - 1.53) |
| Black African | 2.11 (1.14 - 3.90) |
| Black other | 1.28 (0.71 – 2.33) |
| Black overall [a] | 1.64 (1.05 - 2.55) |

Supplementary Table 8. Hazard ratios (HRs) for all-cause mortality using a stringent case-definition for systemic lupus erythematosus (SLE).

Estimates with 95% confidence intervals (95% CI) were obtained using flexible parametric survival models for the overall study period and across time intervals from diagnosis. [1] SLE vs Controls: The control population served as the reference group. Models were adjusted for ethnicity and smoking status, accounting for age and sex through matching. [2] SLE by ethnicity: White ethnicity served as the reference group. Models were adjusted for age, sex, and smoking status.

| | **Overall** | **0-1 year from diagnosis** | **1-5 years from diagnosis** | **5+ years from diagnosis** |
|---|---|---|---|---|
| **SLE vs Controls [1]** | | | | |
| SLE | 2.10 (1.85 - 2.39) | 1.87 (1.34 – 2.60) | 2.08 (1.71 – 2.53) | 2.23 (1.80 – 2.77) |
| **SLE by ethnicity [2]** | | | | |
| White | Reference | Reference | Reference | Reference |
| Asian | 0.84 (0.49 - 1.44) | 0.85 (0.26 - 2.75) | 0.80 (0.38 - 1.68) | 0.78 (0.30 - 2.01) |
| Black | 1.36 (0.77 - 2.41) | 1.98 (0.83 – 4.73) | 0.83 (0.36 - 1.91) | 1.42 (0.68 - 2.97) |

Supplementary Table 9. Age and sex-standardised complication rates (ASCRs) in people with systemic lupus erythematosus (SLE) and matched controls, with 95% confidence intervals (95% CI).

Age and sex-standardised complication rates (ASCRs) are presented per 1,000 person-years and shown separately for people with SLE and matched controls. Low counts (<8) were redacted (*). Abbreviations: IHD, ischaemic heart disease; TIA, transient ischaemic attack; CKD, chronic kidney disease; ILD, interstitial lung disease.

**SLE cohort**

| Complication | Total N | Overall crude rate | Overall ASCR | Male N | Male crude rate | Male ASCR | Female N | Female crude rate | Female ASCR |
|---|---|---|---|---|---|---|---|---|---|
| IHD | 133 | 5.65 (4.99 - 6.39) | 6.41 (5.37 - 7.60) | 38 | 14.73 (11.60 - 18.65) | 13.31 (9.42 - 18.26) | 95 | 4.53 (3.91 - 5.25) | 5.52 (4.46 - 6.74) |
| Heart failure | 156 | 6.35 (5.67 - 7.12) | 7.13 (6.05 - 8.34) | 38 | 13.45 (10.60 - 17.03) | 11.01 (7.79 - 15.12) | 118 | 5.43 (4.76 - 6.19) | 6.47 (5.35 - 7.75) |
| Stroke/TIA | 102 | 4.23 (3.67 - 4.87) | 4.79 (3.91 - 5.82) | 22 | 7.90 (5.74 - 10.82) | 7.66 (4.80 - 11.60) | 80 | 3.75 (3.19 - 4.40) | 4.41 (3.50 - 5.49) |
| Thrombosis | 101 | 4.23 (3.67 - 4.88) | 4.40 (3.59 - 5.35) | 11 | 4.08 (2.56 - 6.40) | 3.73 (1.86 - 6.67) | 90 | 4.25 (3.65 - 4.95) | 4.47 (3.60 - 5.50) |
| Myocarditis/ pericarditis | 72 | 2.95 (2.49 - 3.49) | 3.25 (2.54 - 4.09) | 10 | 3.61 (2.21 - 5.79) | 3.64 (1.74 - 6.69) | 62 | 2.86 (2.38 - 3.44) | 3.20 (2.45 - 4.10) |
| Diabetes | 236 | 10.17 (9.28 - 11.16) | 9.57 (8.39 - 10.87) | 40 | 15.29 (12.12 - 19.24) | 13.28 (9.49 - 18.09) | 196 | 9.52 (8.60 - 10.54) | 9.15 (7.92 - 10.53) |
| CKD | 343 | 15.00 (13.90 - 16.19) | 18.05 (16.19 - 20.06) | 59 | 22.43 (18.56 - 27.06) | 20.41 (15.54 - 26.33) | 284 | 14.04 (12.91 - 15.27) | 17.67 (15.67 - 19.85) |
| CKD stage 5 | 56 | 2.25 (1.86 - 2.74) | 2.35 (1.77 - 3.05) | 15 | 5.18 (3.50 - 7.60) | 5.07 (2.84 - 8.35) | 41 | 1.87 (1.49 - 2.34) | 2.06 (1.47 - 2.79) |
| ILD | 95 | 3.87 (3.34 - 4.48) | 3.98 (3.22 - 4.86) | 18 | 6.35 (4.45 - 8.99) | 5.30 (3.14 - 8.38) | 77 | 3.55 (3.01 - 4.18) | 3.76 (2.97 - 4.70) |
| Osteoporosis | 210 | 8.72 (7.90 - 9.61) | 9.33 (8.11 - 10.68) | 13 | 4.59 (3.00 - 6.93) | 3.87 (2.06 - 6.62) | 197 | 9.26 (8.37 - 10.25) | 10.41 (9.01 - 11.97) |
| Fracture | 280 | 14.27 (13.11 - 15.53) | 15.11 (13.40 - 16.99) | 32 | 15.03 (11.57 - 19.45) | 15.37 (10.51 - 21.70) | 248 | 14.18 (12.96 - 15.51) | 15.05 (13.23 - 17.04) |
| Solid cancer | 101 | 4.15 (3.60 - 4.79) | 4.23 (3.45 - 5.14) | 13 | 4.59 (3.00 - 6.94) | 3.60 (1.92 - 6.16) | 88 | 4.09 (3.51 - 4.77) | 4.28 (3.43 - 5.27) |
| Fibromyalgia | 258 | 10.95 (10.02 - 11.96) | 10.47 (9.23 - 11.82) | * | 1.73 (0.83 - 3.40) | 1.23 (0.40 - 2.87) | * | 12.24 (11.20 - 13.38) | 11.65 (10.26 - 13.18) |

**Control cohort**

| Complication | Total N | Overall crude rate | Overall ASCR | Male N | Male crude rate | Male ASCR | Female N | Female crude rate | Female ASCR |
|---|---|---|---|---|---|---|---|---|---|

| | | | | | | | | | |
|---|---|---|---|---|---|---|---|---|---|
| IHD | 284 | 3.02 (2.78 - 3.28) | 3.03 (2.69 - 3.41) | 76 | 6.89 (5.84 - 8.12) | 5.33 (4.20 - 6.67) | 208 | 2.51 (2.27 - 2.76) | 2.61 (2.27 - 2.99) |
| Heart failure | 282 | 2.92 (2.68 - 3.17) | 3.08 (2.73 - 3.46) | 63 | 5.31 (4.42 - 6.36) | 3.98 (3.06 - 5.10) | 219 | 2.58 (2.35 - 2.84) | 2.88 (2.51 - 3.29) |
| Stroke/TIA | 215 | 2.25 (2.04 - 2.47) | 2.33 (2.03 - 2.67) | 48 | 4.09 (3.31 - 5.04) | 3.16 (2.33 - 4.20) | 167 | 1.99 (1.78 - 2.22) | 2.17 (1.85 - 2.52) |
| Thrombosis | 109 | 1.13 (0.99 - 1.30) | 1.15 (0.94 - 1.39) | 20 | 1.68 (1.20 - 2.33) | 1.21 (0.74 - 1.88) | 89 | 1.06 (0.91 - 1.23) | 1.12 (0.90 - 1.38) |
| Myocarditis/ pericarditis | 23 | 0.24 (0.17 - 0.32) | 0.23 (0.15 - 0.35) | * | 0.42 (0.20 - 0.82) | 0.42 (0.14 - 0.97) | * | 0.21 (0.15 - 0.30) | 0.22 (0.13 - 0.34) |
| Diabetes | 663 | 7.31 (6.93 - 7.72) | 7.02 (6.49 - 7.57) | 110 | 10.21 (8.90 - 11.70) | 8.20 (6.74 - 9.88) | 553 | 6.92 (6.52 - 7.35) | 6.83 (6.27 - 7.42) |
| CKD | 746 | 8.09 (7.68 - 8.51) | 9.44 (8.77 - 10.14) | 140 | 12.41 (11.00 - 14.00) | 10.23 (8.60 - 12.07) | 606 | 7.48 (7.07 - 7.92) | 9.29 (8.56 - 10.06) |
| CKD stage 5 | 26 | 0.27 (0.20 - 0.36) | 0.26 (0.17 - 0.38) | 9 | 0.75 (0.44 - 1.23) | 0.52 (0.24 - 1.00) | 17 | 0.20 (0.14 - 0.29) | 0.21 (0.12 - 0.33) |
| ILD | 36 | 0.37 (0.29 - 0.47) | 0.37 (0.26 - 0.51) | 12 | 0.99 (0.64 - 1.53) | 0.71 (0.37 - 1.24) | 24 | 0.28 (0.21 - 0.38) | 0.30 (0.19 - 0.44) |
| Osteoporosis | 313 | 3.29 (3.04 - 3.56) | 3.48 (3.11 - 3.89) | 21 | 1.75 (1.26 - 2.41) | 1.40 (0.87 - 2.14) | 292 | 3.51 (3.23 - 3.82) | 3.98 (3.54 - 4.47) |
| Fracture | 884 | 11.43 (10.90 - 11.98) | 11.70 (10.95 - 12.50) | 86 | 9.52 (8.15 - 11.12) | 9.45 (7.56 - 11.67) | 798 | 11.68 (11.12 - 12.28) | 12.32 (11.48 - 13.20) |
| Solid cancer | 359 | 3.80 (3.52 - 4.09) | 3.66 (3.29 - 4.06) | 41 | 3.45 (2.74 - 4.33) | 2.43 (1.74 - 3.29) | 318 | 3.85 (3.55 - 4.16) | 3.85 (3.44 - 4.30) |
| Fibromyalgia | 144 | 1.49 (1.33 - 1.68) | 1.35 (1.13 - 1.58) | * | 0.17 (0.05 - 0.48) | 0.29 (0.03 - 1.04) | * | 1.68 (1.49 - 1.90) | 1.50 (1.26 - 1.77) |

Supplementary Table 10. Number of individuals with pre-existing diagnoses excluded from age-standardised complication rate calculations.

Numbers of individuals excluded due to a recorded diagnosis of the outcome prior to the index date are shown for (a) the overall comparison between people with systemic lupus erythematosus (SLE) and matched controls; and (b) analyses restricted to the SLE cohort, in which ethnicity was the exposure of interest. Exclusions were applied symmetrically to cases and controls using full available registration history to ensure that only incident complications were captured during follow-up.

| Complication | SLE v controls model (%) | SLE ethnicity model (%) |
|---|---|---|
| Ischaemic heart disease | 922 (3.7%) | 284 (5.8%) |
| Heart failure | 266 (1.1%) | 97 (2.0%) |
| Stroke/TIA | 512 (2.1%) | 172 (3.5%) |
| Thrombosis | 460 (1.9%) | 210 (4.3%) |
| Myocarditis/pericarditis | 141 (0.6%) | 98 (2.0%) |
| Diabetes | 1,767 (7.2%) | 383 (7.8%) |
| CKD | 1,529 (6.2%) | 433 (8.8%) |
| CKD stage 5 | 47 (0.2%) | 21 (0.4%) |
| ILD | 96 (0.4%) | 73 (1.5%) |
| Osteoporosis | 627 (2.5%) | 182 (3.7%) |
| Fracture | 5,145 (20.9%) | 1,079 (21.9%) |
| Solid cancer | 665 (2.7%) | 109 (2.2%) |
| Fibromyalgia | 493 (2.0%) | 289 (5.9%) |

Supplementary Table 11. Hazard ratios for developing complications of interest in people with systemic lupus erythematosus (SLE) compared with matched controls.

Hazard ratios (HRs) with 95% confidence intervals (95% CI) were estimated using competing risk models. Median time to event is presented in days with interquartile range (IQR). All models were adjusted for ethnicity and accounted for age and sex through matching. Additional covariates were selected based on directed acyclic graphs: * Prior diabetes, prior hypertension, smoking status. ‡ Smoking status, prior oestrogen use. † Smoking status. § Alcohol dependence, smoking status. Abbreviations: TIA, transient ischaemic attack; CKD, chronic kidney disease; ILD, interstitial lung disease.

| Complication | Complication HR (95% CI) | Median time to event, days (IQR) |
|---|---|---|
| Ischaemic heart disease* | 1.77 (1.43 - 2.18) | 1207.0 (409.0,1807.0) |
| Heart failure* | 2.03 (1.67 - 2.48) | 1152.5 (368.5,2199.0) |
| Stroke/TIA* | 1.78 (1.41 - 2.24) | 924.0 (238.0,1937.0) |
| Thrombosis‡ | 3.62 (2.75 - 4.77) | 625.0 (183.0,1533.0) |
| Myocarditis/pericarditis | 10.72 (6.76 - 17.01) | 213.0 (15.0,772.5) |
| Diabetes | 1.26 (1.09 - 1.47) | 1244.5 (526.0,1969.0) |
| CKD* | 1.72 (1.50 - 1.96) | 883.0 (284.0,1861.0) |
| CKD stage 5* | 7.65 (4.75 - 12.34) | 915.5 (338.5,1746.0) |
| ILD† | 9.77 (6.65 - 14.34) | 666.0 (253.0,1926.0) |
| Osteoporosis§ | 2.76 (2.33 - 3.28) | 1065.0 (414.0,1988.0) |
| Fracture§ | 1.29 (1.13 - 1.48) | 1288.5 (579.0,2256.0) |
| Solid cancer† | 1.09 (0.87 - 1.36) | 928.0 (364.0,1987.0) |
| Fibromyalgia | 7.57 (6.13 - 9.34) | 710.0 (280.0,1533.0) |

Supplementary Table 12. Hazard ratios for developing complications of interest in males compared with females.

Hazard ratios (HRs) with 95% confidence intervals (95% CI) were estimated using competing risk models within the systemic lupus erythematosus (SLE) cohort. All models were adjusted for ethnicity and accounted for age through matching. Additional covariates were selected based on directed acyclic graphs: * Prior diabetes, prior hypertension, smoking status. ‡ Smoking status, prior oestrogen use. † Smoking status. § Alcohol dependence, smoking status. Abbreviations: TIA, transient ischaemic attack; CKD, chronic kidney disease; ILD, interstitial lung disease.

| **Complication** | **HR (95%CI)** |
|---|---|
| Ischaemic heart disease* | 2.32 (1.53 - 3.52) |
| Heart failure* | 2.01 (1.36 - 2.96) |
| Stroke/TIA* | 1.62 (0.96 - 2.75) |
| Thrombosis‡ | 1.07 (0.56 - 2.05) |
| Myocarditis/pericarditis | 1.54 (0.80 - 2.97) |
| Diabetes | 1.48 (1.04 - 2.10) |
| CKD* | 1.11 (0.82 - 1.51) |
| CKD stage 5* | 2.80 (1.50 - 5.23) |
| ILD† | 1.65 (0.97 - 2.80) |
| Osteoporosis§ | 0.34 (0.19 - 0.60) |
| Fracture§ | 0.77 (0.52 - 1.13) |
| Solid cancer† | 0.75 (0.40 - 1.38) |
| Fibromyalgia | 0.15 (0.06 - 0.36) |

Supplementary Table 13. Hazard ratios for developing complications of interest by age decile.

Analyses were conducted within the systemic lupus erythematosus (SLE) cohort. Hazard ratios (HRs) with 95% confidence intervals (95% CI) represent the relative change in risk per one-unit increase in age decile, with age modelled as a continuous variable. Estimates were obtained using competing risk models. All models were adjusted for ethnicity, with sex accounted for through matching. Additional covariates were selected based on directed acyclic graphs: * Prior diabetes, prior hypertension, smoking status. ‡ Smoking status, prior oestrogen use. † Smoking status. § Alcohol dependence, smoking status. Abbreviations: TIA, transient ischaemic attack; CKD, chronic kidney disease; ILD, interstitial lung disease.

| **Complication** | **HR (95% CI)** |
|---|---|
| Ischaemic heart disease* | 1.51 (1.32 - 1.73) |
| Heart failure* | 1.67 (1.45 - 1.92) |
| Stroke/TIA* | 1.21 (1.02 - 1.44) |
| Thrombosis‡ | 1.05 (0.91 - 1.20) |
| Myocarditis/pericarditis | 0.76 (0.64 - 0.91) |
| Diabetes | 1.22 (1.13 - 1.31) |
| CKD* | 1.45 (1.32 - 1.60) |
| CKD stage 5* | 0.75 (0.59 - 0.95) |
| ILD† | 1.34 (1.19 - 1.52) |
| Osteoporosis§ | 1.71 (1.57 - 1.87) |
| Fracture§ | 1.26 (1.16 - 1.36) |
| Solid cancer† | 1.58 (1.39 - 1.79) |
| Fibromyalgia | 0.81 (0.76 - 0.87) |

Supplementary Table 14. Solid and haematological cancer outcomes in people with systemic lupus erythematosus (SLE) and matched controls.

Total numbers (N), age and sex-standardised complication rates (ASCRs), and hazard ratios (HRs) with 95% confidence intervals (95% CI) are shown for incident solid and haematological cancers in the SLE and control cohorts. ASCRs are presented per 1,000 person-years and shown separately for people with SLE and matched controls. Hazard ratios were estimated using competing risk models adjusted for ethnicity and smoking status, with age and sex accounted for through matching. Individuals at risk were defined separately for each outcome, excluding participants with a recorded diagnosis of that cancer subtype prior to the index date.

| Complication | SLE total (N) | SLE individuals at risk (N) | Control total (N) | Control individuals at risk (N) | SLE ASCR (95% CI) | Control ASCR (95% CI) | HR (95% CI) |
|---|---|---|---|---|---|---|---|
| Solid cancers | 101 | 4,828 | 359 | 19,151 | 4.23 (3.45 - 5.14 | 3.66 (3.29 - 4.06) | 1.09 (0.87 - 1.36) |
| Haematological cancers | 35 | 4,914 | 58 | 19,652 | 1.53 (1.07 - 2.13) | 0.57 (0.43 - 0.74) | 2.36 (1.53 - 3.64) |

Supplementary Table 15. Age and sex-standardised complication rates (ASCRs) per 1,000 person-years in people with systemic lupus erythematosus (SLE) by ethnicity, with 95% confidence intervals (95% CI).

Age-standardised complication rates are presented per 1,000 person-years and stratified by ethnicity within the SLE cohort. Individuals from Mixed/Other or unknown ethnic groups are not shown. Low counts (<8) were redacted (*).

**SLE cohort**

| | White | | | Asian | | | Black | | |
|---|---|---|---|---|---|---|---|---|---|
| **Complication** | **N** | **Crude rate** | **ASCR** | **N** | **Crude rate** | **ASCR** | **N** | **Crude rate** | **ASCR** |
| IHD | 115 | 6.69 (5.86 - 7.65) | 6.75 (5.57 - 8.10) | 9 | 2.98 (1.76 - 4.90) | 4.12 (1.88 - 7.81) | * | 2.04 (0.98 - 4.01) | 3.14 (1.02 - 7.34) |
| Heart failure | 126 | 6.99 (6.16 - 7.94) | 6.75 (5.62 - 8.03) | 10 | 3.20 (1.96 - 5.14) | 7.14 (3.42 - 13.13) | 15 | 5.99 (4.04 - 8.78) | 6.41 (3.59 - 10.57) |
| Stroke/TIA | 83 | 4.71 (4.02 - 5.52) | 4.84 (3.85 - 6.00) | * | 1.61 (0.77 - 3.18) | 1.68 (0.55 - 3.93) | 12 | 4.79 (3.07 - 7.36) | 7.39 (3.82 - 12.91) |
| Thrombosis | 69 | 3.96 (3.33 - 4.71) | 3.90 (3.03 - 4.93) | 8 | 2.59 (1.48 - 4.41) | 2.27 (0.98 - 4.47) | 19 | 7.80 (5.52 - 10.94) | 8.08 (4.86 - 12.62) |
| Myocarditis/pericarditis | 35 | 1.95 (1.52 - 2.50) | 2.29 (1.59 - 3.18) | 8 | 2.55 (1.46 - 4.33) | 2.19 (0.95 - 4.31) | 29 | 11.92 (9.05 - 15.64) | 10.81 (7.24 - 15.52) |
| Diabetes | 148 | 8.63 (7.67 - 9.70) | 7.93 (6.70 - 9.31) | 48 | 16.70 (13.53 - 20.58) | 20.55 (15.15 - 27.24) | 38 | 16.60 (13.08 - 21.02) | 17.91 (12.68 - 24.59) |
| CKD | 256 | 15.41 (14.10 - 16.83) | 16.92 (14.91 - 19.12) | 42 | 13.72 (10.95 - 17.17) | 18.14 (13.07 - 24.52) | 33 | 14.24 (11.01 - 18.36) | 16.25 (11.19 - 22.82) |
| CKD stage 5 | 35 | 1.92 (1.49 - 2.45) | 1.97 (1.37 - 2.74) | * | 2.23 (1.22 - 3.94) | 4.31 (1.73 - 8.89) | 12 | 4.80 (3.08 - 7.38) | 6.38 (3.29 - 11.14) |
| ILD | 60 | 3.32 (2.75 - 4.00) | 3.05 (2.33 - 3.93) | 14 | 4.51 (3.00 - 6.72) | 4.64 (2.54 - 7.79) | 19 | 7.73 (5.47 - 10.85) | 8.88 (5.35 - 13.87) |
| Osteoporosis | 166 | 9.43 (8.44 - 10.53) | 8.91 (7.60 - 10.37) | 27 | 8.82 (6.62 - 11.69) | 17.14 (11.30 - 24.94) | 13 | 5.18 (3.38 - 7.83) | 9.23 (4.91 - 15.78) |
| Fracture | 222 | 16.18 (14.71 - 17.80) | 15.35 (13.40 - 17.51) | 35 | 12.15 (9.47 - 15.54) | 17.58 (12.24 - 24.44) | 18 | 8.02 (5.62 - 11.36) | 9.65 (5.72 - 15.25) |
| Solid cancer | 83 | 4.67 (3.98 - 5.46) | 4.21 (3.35 - 5.22) | * | 2.25 (1.23 - 3.97) | 6.17 (2.48 - 12.71) | * | 2.79 (1.53 - 4.93) | 3.62 (1.45 - 7.45) |
| Fibromyalgia | 202 | 11.77 (10.65 - 13.01) | 11.57 (10.03 - 13.28) | 28 | 9.21 (6.96 - 12.15) | 8.41 (5.59 - 12.15) | 16 | 6.52 (4.46 - 9.45) | 5.58 (3.19 - 9.06) |

**Control cohort**

| | White | | | Asian | | | Black | | |
|---|---|---|---|---|---|---|---|---|---|
| **Complication** | **N** | **Crude rate** | **ASCR** | **N** | **Crude rate** | **ASCR** | **N** | **Crude rate** | **ASCR** |

| | | | | | | | | | |
|---|---|---|---|---|---|---|---|---|---|
| IHD | 242 | 3.20 (2.92 - 3.50) | 2.97 (2.61 - 3.37) | 22 | 2.51 (1.83 - 3.44) | 3.68 (2.30 - 5.56) | 13 | 2.70 (1.77 - 4.09) | 4.82 (2.57 - 8.25) |
| Heart failure | 247 | 3.17 (2.89 - 3.47) | 3.01 (2.65 - 3.41) | 13 | 1.45 (0.95 - 2.19) | 4.22 (2.25 - 7.21) | 13 | 2.67 (1.75 - 4.04) | 4.55 (2.42 - 7.78) |
| Stroke/TIA | 191 | 2.48 (2.23 - 2.74) | 2.37 (2.05 - 2.73) | 15 | 1.67 (1.13 - 2.45) | 2.71 (1.52 - 4.46) | * | 1.45 (0.79 - 2.56) | 3.00 (1.21 - 6.19) |
| Thrombosis | 93 | 1.20 (1.03 - 1.39) | 1.14 (0.92 - 1.39) | * | 0.67 (0.35 - 1.24) | 0.89 (0.33 - 1.93) | * | 1.47 (0.80 - 2.59) | 1.40 (0.56 - 2.89) |
| Myocarditis/pericarditis | 20 | 0.26 (0.18 - 0.35) | 0.25 (0.15 - 0.38) | * | 0.33 (0.12 - 0.80) | 0.39 (0.08 - 1.13) | 0 | - | - |
| Diabetes | 491 | 6.65 (6.24 - 7.09) | 6.03 (5.51 - 6.59) | 104 | 13.33 (11.58 - 15.34) | 21.64 (17.68 - 26.22) | 52 | 11.85 (9.68 - 14.48) | 16.37 (12.22 - 21.46) |
| CKD | 605 | 8.14 (7.69 - 8.62) | 8.79 (8.10 - 9.51) | 63 | 7.35 (6.12 - 8.82) | 13.79 (10.59 - 17.64) | 54 | 11.59 (9.51 - 14.11) | 22.64 (17.00 - 29.53) |
| CKD stage 5 | 20 | 0.25 (0.18 - 0.35) | 0.23 (0.14 - 0.35) | * | 0.33 (0.12 - 0.80) | 0.69 (0.14 - 2.01) | * | 0.61 (0.23 - 1.48) | 1.35 (0.28 - 3.96) |
| ILD | 30 | 0.38 (0.29 - 0.50) | 0.34 (0.23 - 0.49) | * | 0.55 (0.27 - 1.09) | 0.73 (0.24 - 1.71) | 0 | - | - |
| Osteoporosis | 289 | 3.78 (3.48 - 4.11) | 3.60 (3.20 - 4.04) | 13 | 1.45 (0.95 - 2.19) | 3.56 (1.90 - 6.09) | * | 1.23 (0.64 - 2.28) | 1.96 (0.72 - 4.26) |
| Fracture | 779 | 12.91 (12.28 - 13.58) | 12.54 (11.67 - 13.45) | 53 | 6.40 (5.24 - 7.80) | 9.13 (6.84 - 11.94) | 20 | 4.42 (3.16 - 6.15) | 5.38 (3.29 - 8.31) |
| Solid cancer | 318 | 4.18 (3.86 - 4.53) | 3.73 (3.33 - 4.16) | 19 | 2.13 (1.51 - 2.99) | 3.15 (1.90 - 4.92) | 12 | 2.49 (1.60 - 3.83) | 3.37 (1.74 - 5.88) |
| Fibromyalgia | 117 | 1.51 (1.32 - 1.72) | 1.40 (1.15 - 1.67) | 17 | 1.90 (1.31 - 2.72) | 1.66 (0.97 - 2.66) | * | 0.82 (0.36 - 1.76) | 0.55 (0.15 - 1.40) |

Supplementary Table 16. Hazard ratios for developing complications of interest in people with systemic lupus erythematosus (SLE) by ethnicity.

Hazard ratios (HRs) with 95% confidence intervals (95% CI) were estimated within the SLE cohort using competing risk models. White ethnicity served as the reference group. Models were adjusted for age, sex, with additional covariates based on directed acyclic graphs: * Prior diabetes, prior hypertension, smoking status. ‡ Smoking status, prior oestrogen use. † Smoking status. § Alcohol dependence, smoking status. Abbreviations: TIA, transient ischaemic attack; CKD, chronic kidney disease; ILD, interstitial lung disease.

| **Complication** | **White** | **Asian HR (95% CI)** | **Black HR (95% CI)** |
|---|---|---|---|
| Ischaemic heart disease* | Reference | 0.81 (0.40 - 1.64) | 0.54 (0.22 - 1.37) |
| Heart failure* | Reference | 0.75 (0.39 - 1.45) | 1.50 (0.87 - 2.59) |
| Stroke/TIA* | Reference | 0.43 (0.17 - 1.08) | 1.28 (0.67 - 2.46) |
| Thrombosis‡ | Reference | 0.68 (0.33 - 1.44) | 2.10 (1.24 - 3.55) |
| Myocarditis/pericarditis | Reference | 1.01 (0.46 - 2.23) | 4.99 (2.97 - 8.39) |
| Diabetes | Reference | 2.55 (1.75 - 3.72) | 2.55 (1.73 - 3.76) |
| CKD* | Reference | 1.23 (0.87 - 1.76) | 1.35 (0.96 - 1.89) |
| CKD stage 5* | Reference | 0.86 (0.38 - 1.96) | 1.89 (0.97 - 3.69) |
| ILD† | Reference | 1.72 (0.94 - 3.12) | 3.14 (1.81 - 5.47) |
| Osteoporosis§ | Reference | 1.46 (0.93 - 2.29) | 0.93 (0.51 - 1.70) |
| Fracture§ | Reference | 1.05 (0.72 - 1.54) | 0.60 (0.35 - 1.03) |
| Solid cancer† | Reference | 0.82 (0.36 - 1.86) | 1.00 (0.46 - 2.19) |
| Fibromyalgia | Reference | 0.67 (0.45 - 0.98) | 0.44 (0.26 - 0.73) |

Supplementary Table 17. Hazard ratios for developing myocarditis/pericarditis, interstitial lung disease (ILD) or thrombosis in Black patients with systemic lupus erythematosus (SLE).

Hazard ratios (HRs) with 95% confidence intervals (95% CI) were estimated within the SLE cohort using competing risk models. White ethnicity served as the reference group. Models were adjusted for age and sex, with additional covariates based on directed acyclic graphs: † Smoking status. ‡ Smoking status, prior oestrogen use.

| | | **Hazard ratios** | |
|---|---|---|---|
| | **Ethnicity** | **0-1 year from diagnosis** | **1+ years from diagnosis** |
| Myocarditis/pericarditis | White | Reference | Reference |
| | Black | 5.50 (2.85 - 10.63) | 4.07 (1.73 - 9.54) |
| ILD† | White | Reference | Reference |
| | Black | 4.18 (1.71 - 10.21) | 2.75 (1.34 - 5.63) |
| Thrombosis‡ | White | Reference | Reference |
| | Black | 2.19 (0.94 - 5.10) | 2.06 (1.02 - 4.14) |
